# Competition Between C_α_‐S and C_α_‐C_β_ Bond Cleavage in β‐Hydroxysulfoxides Cation Radicals Generated by Photoinduced Electron Transfer^†^

**DOI:** 10.1111/php.13455

**Published:** 2021-06-03

**Authors:** Andrea Lapi, Claudio D'Alfonso, Tiziana Del Giacco, Osvaldo Lanzalunga

**Affiliations:** ^1^ Dipartimento di Chimica Universita` degli Studi di Roma “La Sapienza” Rome Italy; ^2^ Istituto per i Sistemi Biologici (ISB‐CNR) Sede Secondaria di Roma‐Meccanismi di Reazione c/o Dipartimento di Chimica Universita` degli Studi di Roma “La Sapienza” Rome Italy; ^3^ Dipartimento di Chimica, Biologia e Biotecnologie Università di Perugia Perugia Italy; ^4^ Centro di Eccellenza Materiali Innovativi Nanostrutturati (CEMIN) Università di Perugia Perugia Italy; ^5^ Present address: Department of Public Security Central Anticrime Directorate of Italian National Police Forensic Science Police Service (DAC‐SPS) Psychotropic and Narcotic Substances Section Rome Italy

## Abstract

A kinetic and product study of the 3‐cyano‐*N*‐methyl‐quinolinium photoinduced monoelectronic oxidation of a series of β‐hydroxysulfoxides has been carried out to investigate the competition between C_α_‐S and C_α_‐C_β_ bond cleavage within the corresponding cation radicals. Laser flash photolysis experiments unequivocally established the formation of sulfoxide cation radicals showing their absorption band (*λ*_max_ ≈ 520 nm) and that of 3‐CN‐NMQ^•^ (*λ*_max_ ≈ 390 nm). Steady‐state photolysis experiments suggest that, in contrast to what previously observed for alkyl phenyl sulfoxide cation radicals that exclusively undergo C_α_‐S bond cleavage, the presence of a β‐hydroxy group makes, in some cases, the C_α_‐C_β_ scission competitive. The factors governing this competition seem to depend on the relative stability of the fragments formed from the two bond scissions. Substitution of the β‐OH group with ‐OMe did not dramatically change the reactivity pattern of the cation radicals thus suggesting that the observed favorable effect of the hydroxy group on the C_α_‐C_β_ bond cleavage mainly resides on its capability to stabilize the carbocation formed upon this scission.

## Graphical abstract

The behavior of a series of β‐hydroxysulfoxide cation radicals, obtained by photoinduced electron transfer, have been studied via laser flash photolysis and steady state experiments with the aim to investigate the competition of Cα‐S and Cα‐Cβ bond cleavage.

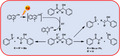

## INTRODUCTION

It is a great honor for us for having been invited to contribute to the Special Issue of *Photochemistry & Photobiology* dedicated to celebrating the career of Dr. Edward Clennan. Because of Ed’s fundamental contribution to the photochemistry of sulfur‐containing compounds, it is a pleasure to present here our work concerning the one‐electron photooxidation of β‐hydroxysulfoxides promoted by 3‐cyano‐*N*‐methyl‐quinolinium.

It is well known that electron transfer (ET) processes play a fundamental role in many biological and organic processes. For this reason, an increasing number of studies have been focused on the reactivity and the properties of the radical ions, the primary species obtained from these processes (1, 2). Among the classes of organic compounds whose reactivity in ET process have been the subject of intense investigation, sulfides have attracted a special interest (3, 4, 5, 6, 7, 8) since their monoelectronic oxidation is involved in many biological processes (9), in organic synthesis (10) and in the initiation of radical polymerization (11). To better elucidate the reaction mechanisms involved in sulfides oxidation initiated by an ET process, the use of photosensitizers as initiators has been widely employed particularly in type I (12) sulfide photooxygenation (6, 13, 14). Despite the large number of studies on the monoelectronic oxidation of organic sulfides, the same process on their oxidized form, organic sulfoxides, is much less investigated albeit, in the past decades, the comprehension of their reactivity and properties has attracted the interest of several research groups since sulfoxides are involved in many synthetic and biological processes (15, 16, 17, 18, 19). The main reason for the scarce information on the reactivity and properties of sulfoxide cation radicals is due to the fact that sulfoxides generally exhibit redox potentials 0.5 V higher than those of the corresponding sulfides (20, 21) making them less prone to undergo monoelectronic oxidation. As an example, the redox potentials of methyl phenyl sulfoxide and thioanisole are 2.01 (21) and 1.47 V (22) (*vs* SCE), respectively.

**Scheme 1:**
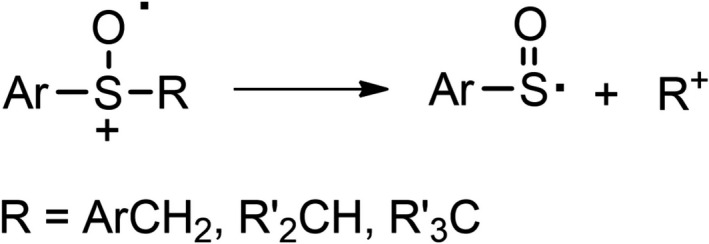
C_α_‐S bond cleavage in alkyl aryl sulfoxide cation radicals.

Previous studies on the reactivity of alkyl aryl sulfoxide cation radicals (ArSOR^+•^) have shown that the fate of these oxidized species largely depends on the nature of the alkyl substituent R. When R is a methyl or a primary alkyl group (except the benzyl group), once formed by photosensitized oxidation, the sulfoxide cation radical undergoes unproductive back electron transfer (BET) (21). When R is a benzylic, secondary or tertiary alkyl group, the cation radical undergoes C_α_‐S bond cleavage affording the R^+^ carbocation and the phenyl sulfinyl radical ArSO^•^ (Scheme 1) (23, 24, 25).

The C_α_‐S bond cleavage is a peculiar process characterizing both sulfide and sulfoxide cation radicals (23, 24, 25, 26, 27, 28, 29, 30, 31, 32, 33, 34) since the fragmentation of cation radicals of other classes of organic compounds often involves the bonds in β position with respect to the charged center. In a previous study, on the reactivity of aryl sulfide cation radicals bearing a hydroxy group in β position, we showed that the OH group determines the C_α_‐C_β_ instead of the C_α_‐S bond cleavage (35). The favorable effect of the hydroxy group on the C_α_‐C_β_ bond cleavage was proposed to be due to a transition state stabilization by a hydrogen bonding between the OH and the solvent (MeCN). In view of the above‐mentioned higher redox potential of sulfoxides with respect to sulfides and of the higher stability of the phenyl sulfinyl radical PhSO^•^ as compared to the phenylthiyl radical PhS^•^ (36), the C_α_‐S bond cleavage is very much faster for sulfoxide cation radicals rather than for sulfide ones (24, 35), thus competition between C_α_‐S and C_α_‐C_β_ bond cleavage for β‐hydroxysulfoxides cation radicals can be foreseen. On these bases, we now report a kinetic and product study of the fragmentation process of a series of β‐hydroxysulfoxide (Chart 1) cation radicals. To better understand the role played by the β‐hydroxy group, the investigation was also extended to two β‐methoxysulfoxides (**5** and **6**).

**Chart Chart 1:**
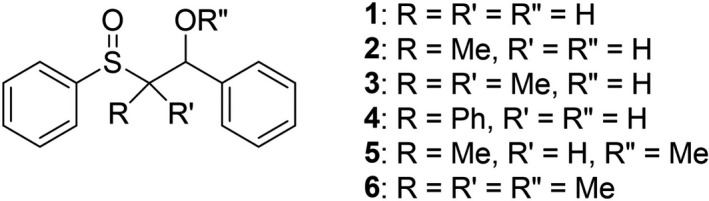
β‐hydroxy and β‐methoxy sulfoxides investigated in this work.

**Scheme 2:**
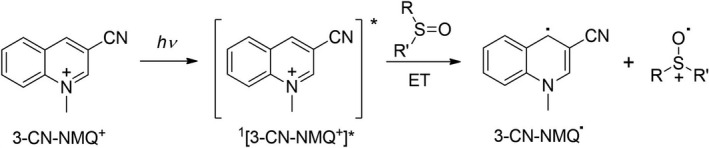
Photosensitized monoelectronic oxidation of sulfoxides promoted by 3‐cyano‐*N*‐methyl‐quinolinium.

The cation radicals were generated by photosensitized monoelectronic oxidation of the parent β‐hydroxysulfoxides as already reported in previous studies on the reactivity of alkyl aryl sulfoxide cation radicals (21, 23, 24, 25). The photosensitizer chosen for this purpose was the 3‐cyano‐*N*‐methyl‐quinolinium perchlorate (3‐CN‐NMQ^+^ ClO_4_^−^, Scheme 2) for the following reasons: (1) it is a powerful oxidant in its excited singlet state having a reduction potential of 2.72 V *vs* SCE in MeCN (37); (2) the ^1^[3‐CN‐NMQ^+^]* lifetime is sufficiently long (*τ* = 45 ns) (37) to efficiently interact with the substrates investigated; (3) the UV absorption maximum of 3‐CN‐NMQ^+^ at ca 330 nm allows a selective photosensitizer excitation thus avoiding a direct photolysis of the sulfoxides **1**–**6** which absorb below 320 nm. Finally, since the photosensitizer is positively charged, upon the ET process a cation radical/radical couple is formed with a consequent easier separation of the two species (lack of electrostatic barrier) thus depressing the unproductive BET process.

## MATERIALS AND METHODS

### Chemicals and instrumentation

^1^H and ^13^C NMR spectra were recorded on a Bruker Avance spectrometer at 300 and 75 MHz, respectively, in CD_3_CN or CDCl_3_ (Sigma‐Aldrich) and using tetramethylsilane as the internal standard. GC‐MS analyses were performed on an HP 5890 series II GC equipped with an HP‐5 capillary column (30 m × 0.25 mm × 0.25 μm) and coupled with an HP 5972 MSD mass spectrometer. HPLC analyses were performed on an Agilent 1100 chromatograph coupled with a UV‐Vis detector (set at 263 nm wavelength) and equipped with an Alltima C18 column (5 μ, 250 mm × 4.6 mm), equilibrated with MeOH/H_2_O (75/25), with an ascending gradient of MeOH (75 − 100%) at a flow rate of 0.7 mL min^−1^. GC analyses were performed on an Agilent series II instrument equipped with an HP‐1 column (30 m × 0.32 mm × 0.25 μm). Spectrophotometric analyses were carried out on a Varian Cary 300 Bio double ray spectrophotometer. FT‐IR spectra were recorded on a Shimadzu FTIR‐8400S infrared spectrophotometer. Steady‐state photoreactions were carried out on a Helios Italquartz photoreactor equipped with four external fluorescence lamps (14 W each) with emission centered at 360 nm and carried out in jacketed Pyrex tubes thermostated using a water circulation Haake F3 thermostat.

All the solvents used were commercially available at the highest purity. Acetonitrile (spectrometric grade) was distilled over CaH_2_ prior use. THF was refluxed over sodium and then distilled under inert (Ar) atmosphere.

3‐Cyano‐*N*‐methylquinolinium perchlorate (38) and 1‐phenyl‐2‐phenylsulfinyl‐2‐methylpropan‐1‐ol (**3**) were synthesized according to a previously reported procedure (39). 1,2‐Diphenyl‐2‐phenylsulfinyl ethanol (**4**) was synthesized by oxidation of the corresponding sulfide (1,2‐diphenyl‐2‐phenylsulfanyl ethanol) (35), with NaIO_4_ as described elsewhere (40).

#### Synthesis of 1‐phenyl‐2‐phenylsulfinyl ethanol (1)

A solution of thiophenol (4.4 g, 40 mmol) in acetone (20 mL) was slowly added in a 500 mL three‐necked round‐bottomed flask containing an Ar‐saturated stirred mixture of α‐bromoacetophenone (8.0 g, 40 mmol) and anhydrous K_2_CO_3_ (6.1 g, 44 mmol) in acetone (250 mL) at room temperature. The mixture was stirred for 6 h, quenched by addition of HCl (2 m) until complete carbonate dissolution and extracted with three portions of diethyl ether (100 mL each). The collected ethereal phases were washed with saturated NaHCO_3_, dried over anhydrous Na_2_SO_4_ and concentrated at reduced pressure. Recrystallization from hexane afforded pure (> 99%, GC) 1‐phenyl‐2‐phenylsulfanylethanone (5.1 g, 22.4 mmol, 56% yield) as a white solid. ^1^H NMR (CDCl_3_) δ (ppm): 7.95‐7.93 (m, 2H), 7.48‐7.24 (m, 8H), 4.27 (s, 2H), in agreement with that reported in ref. (41). 1‐Phenyl‐2‐phenylsulfanylethanone was then converted to 1‐phenyl‐2‐phenylsulfanyl ethanol by reduction with NaBH_4_ as reported in ref. (42). 1‐Phenyl‐2‐phenylsulfanyl ethanol was then converted to the title compound as follows. NaIO_4_ (1.3 g, 6.3 mmol) was added to a solution of 1‐phenyl‐2‐phenylsulfanyl ethanol (1.3 g, 5.7 mmol) in 5:1 EtOH/H_2_O (60 mL) and stirred for 24 h at room temperature. The mixture was concentrated under reduced pressure and extracted with three portions of ethyl acetate (20 mL each). The collected organic phases were dried over anhydrous Na_2_SO_4_ and concentrated under reduced pressure. Column chromatography purification (silica gel, hexane/ethyl acetate 1:1) afforded pure (> 99%, HPLC) 1‐phenyl‐2‐phenylsulfinyl ethanol (1.3 g, 93%) as a white solid. ^1^H and ^13^C NMR analysis agreed with those reported in ref. (43). Major diastereomer (ca 60%): ^1^H NMR (CDCl_3_) δ (ppm): 7.68–7.27 (m, 10H), 5.27 (d, 1H, *J* = 10 Hz), 4.16 (s, 1H), 3.33–3.19 (m, 1H), 2.86 (d, 1H, *J* = 13 Hz). ^13^C NMR (CDCl_3_) δ (ppm): 132.2, 131.9, 130.1, 129.3, 128.8, 128.6, 126.4, 126.2, 124.7, 124.6, 71.7, 69.3.

Minor diastereomer (ca 40%): ^1^H NMR (CDCl_3_) δ (ppm): 7.68‐7.27 (m, 10H), 5.42 (d, 1H, *J* = 10 Hz), 4.28 (s, 1H), 3.33‐3.19 (m, 1H), 2.98 (dd, 1H, *J* = 13 Hz, *J* = 2.5 Hz).

#### Synthesis of 1‐phenyl‐2‐phenylsulfinylpropan‐1‐ol (2)

The title compound was obtained by oxidation of the corresponding sulfide 1‐phenyl‐2‐phenylsulfanylpropan‐1‐ol prepared by reaction of cis‐β‐methylstyrene oxide with thiophenol according to ref. (44). The sulfoxidation procedures are the same as reported above for **1** using 1‐phenyl‐2‐phenylsulfanylpropan‐1‐ol (1.0 g, 4.1 mmol) and NaIO_4_ (1.0 g, 4.7 mmol) as starting material. After the workup procedure pure (> 99%, HPLC) 1‐phenyl‐2‐phenylsulfinylpropan‐1‐ol (826 mg, 3.2 mmol, 78%) was obtained as a white solid. ^1^H and ^13^C NMR analysis are in agreement with those reported in ref. (45).

^1^H NMR (CD_3_CN) δ (ppm): 7.45‐7.05 (m, 10H), 4.70 (dq, 1H, *J* = 6.45 Hz, *J* = 2.94 Hz), 3.63 (d, 1H, *J* = 2.94 Hz), 1.03 (d, 3H, *J* = 6.45 Hz).

^13^C NMR (CD_3_CN) δ (ppm): 131.9, 131.8, 131.6, 131.5, 129.5, 129.1, 128.7, 128.4, 126, 125.4, 78.7, 65.0, 21.6.

#### Synthesis of [(1‐mehoxy‐1‐phenylpropyl)sulfinyl]benzene (5)

To a stirred suspension of NaH (281 mg, 12 mmol) in anhydrous THF (10 mL) 1‐phenyl‐2‐phenylsulfanylpropan‐1‐ol (1.2 g, 4.9 mmol) was slowly added under an Ar atmosphere. After 30 min stirring at room temperature, CH_3_I (1.7 g, 12 mmol) was slowly added and the mixture was then allowed to react overnight. After water addition (50 mL), the mixture was concentrated at reduced pressure, solubilized in diethyl ether (100 mL), washed twice with water (50 mL), dried over anhydrous Na_2_SO_4_ and concentrated at reduced pressure. Column chromatography (silica gel, hexane/ethyl acetate 10:1) afforded pure (>99% GC) [(1‐mehoxy‐1‐phenylpropyl)sulfanyl]benzene (1.2 g, 4.7 mmol, 95%). The β‐methoxysulfide (1.2 g, 4.7 mmol) was then oxidized with NaIO_4_ (1.1 g, 5.3 mmol) in ethanol following the same procedure reported above for **2**. Pure (>99%, HPLC) [(1‐mehoxy‐1‐phenylpropyl)sulfinyl]benzene (1.1 g, 3.9 mmol, 83%) was obtained.

Major diastereomer (ca 60%): ^1^H NMR (CDCl_3_) δ (ppm): 7.46–7.04 (m, 10H), 4.42 (m, 1H), 3.58 (s, 3H), 3.54 (d, 1H, *J* = 3.2 Hz), 1.08 (d, 3H, *J* = 6.6 Hz). ^13^C NMR (CDCl_3_) δ (ppm): 131.1, 130.9, 130.3, 128.5, 128.1, 125.6, 80.1, 73.7, 58.0, 16.8.

Minor diastereomer (ca 40%): ^1^H NMR (CDCl_3_) δ (ppm): 7.46‐7.04 (m, 10H), 4.42 (m, 1H), 3.58 (s, 3H), 3.54 (d, 1H, *J* = 3.2 Hz), 1.07 (d, 3H, *J* = 6.6 Hz).

FT‐IR: υ (cm^−1^) 1038 (S = O), 1097 (C‐O‐C), 1130 (C‐O‐C).

#### Synthesis of [(1‐methoxy‐2‐methyl‐1‐phenylpropyl)sulfinyl]benzene (6)

To a stirred suspension of NaH (70 mg, 3 mmol) in anhydrous THF (10 mL) 2‐methyl‐1‐phenyl‐2‐phenylsulfinylpropan‐1‐ol (**3**) (300 mg, 1.2 mmol) was slowly added under an Ar atmosphere. After 30 min stirring at room temperature, CH_3_I (426 mg, 3 mmol) was slowly added and the mixture was stirred for 3 h. After water addition (20 mL), the mixture was concentrated at reduced pressure, solubilized in chloroform (100 mL), washed twice with water (50 mL), dried over anhydrous Na_2_SO_4,_ and concentrated at reduced pressure. Recrystallization from hexane afforded pure (>99%, HPLC) as a white solid (316 mg, 1.1 mol 92%).

^1^H NMR (CDCl_3_) δ (ppm): 7.64–7.26 (m, 10H), 4.65 (s, 1H), 3.36 (s, 3H), 0.98 (s, 3H), 0.68 (s, 3H).

^13^C NMR (CDCl_3_) δ (ppm): 137.4, 131.8, 129.3, 129.1, 128.8, 128.7, 127.5, 83.9, 65.1, 58.1, 16.4, 15.5.

FT‐IR: υ (cm^−1^) 1038 (S=O), 1074 (C‐O‐C), 1095 (C‐O‐C).

### Cyclic Voltammetry

Anodic peak potential values ($E_{p}^{a}$) for **1**–**6** were obtained by cyclic voltammetry experiments, conducted with a potentiostat controlled by a programmable function generator (cyclic voltammetry at 500 mV s^−1^, 1.5 mm diameter glassy carbon disk anode, 1 cm^2^ platinum counter electrode and Ag/AgCl (KCl 3 m) as reference) in Ar‐saturated anhydrous MeCN−Bu_4_NBF_4_ (0.1 m) at 25 °C. Sulfoxide concentration was 2 mm.

### Fluorescence quenching

Measurements were carried out at 25 °C on a Shimadzu RF5001PC spectrofluorophotometer. For quenching of ^1^[3‐CN‐NMQ^+^]* by **1**‐**5**, a solution of 3‐CN‐NMQ^+^ (1.7 × 10^−5^ m) and **1**‐**5** at variable concentration (from 0 to 1 × 10^−2^ m) in argon‐saturated acetonitrile was irradiated at 330 nm (3‐CN‐NMQ^+^ absorption maximum) collecting the relative emission intensities at 425 nm (3‐CN‐NMQ^+^ emission maximum).

### Laser flash photolysis

Nanosecond laser flash photolysis experiments were carried out with a laser kinetic spectrometer using the third harmonic (355 nm) of a Q‐switched Nd:YAG laser delivering 7 ns pulses. The laser energy was < 3 mJ per pulse. In all the experiments, a 3 mL quartz cell containing a solution of **1**–**6** (1.0 × 10^−2^ m), 3‐CN‐NMQ^+^ClO_4_^−^ (0.5 × 10^−4^ m), and the cosensitizer toluene (1 m) in N_2_‐saturated MeCN was flashed at 22 ± 2 °C. The transient spectra were obtained by a point‐to‐point technique, monitoring the ΔA values after the laser flash at 5–10 nm intervals, averaging at least 10 decays at each wavelength. The estimated error for the decay rate constants was ± 10%.

### Photooxidation general procedure

Photooxidation reactions were carried out irradiating (10 or 30 min) an Ar‐saturated MeCN (5 mL) stirred solution of the sulfoxide (50 μmol) and 3‐CN‐NMQ^+^ ClO_4_^−^ (10 μmol) placed in a rubber cap‐sealed jacketed pyrex tube thermostated at 25 °C by water circulation. Bibenzyl (10 μmol) was added as the internal standard. The reaction mixture was analyzed by HPLC, GC, GC‐MS and (after solvent evaporation under reduced pressure) ^1^H NMR. Blank experiments were carried out under the same reaction condition reported above but in the absence of photosensitizer.

### Product analysis

Qualitative analyses were performed by GC‐MS and ^1^H NMR whereas quantitative product determination was carried out by ^1^H NMR, GC and HPLC by comparison with authentic specimens. Quantitative determination of diphenyl disulfide and phenyl phenylthiosulfonate was exclusively carried out by HPLC. Benzaldehyde, diphenyl disulfide, benzyl methyl ketone and diphenylacetaldehyde were commercially available. Phenyl phenylthiosulfonate was prepared as previously reported (46). 2‐Methoxy‐1‐phenylpropan‐1‐ol (47, 48) and 2‐methyl‐2‐phenylpropanal (49) were identified by comparison with spectral properties (^1^H NMR and GC‐MS) reported in the literature.

## RESULTS AND DISCUSSION

The sulfoxides investigated in this work contain two (**1** and **3**) or three (**2**, **4**, **5** and **6**) chiral centers. The following results are thus referred to the stereoisomers mixture for compounds **1**–**6**.

### Electrochemical properties

**Figure 1:**
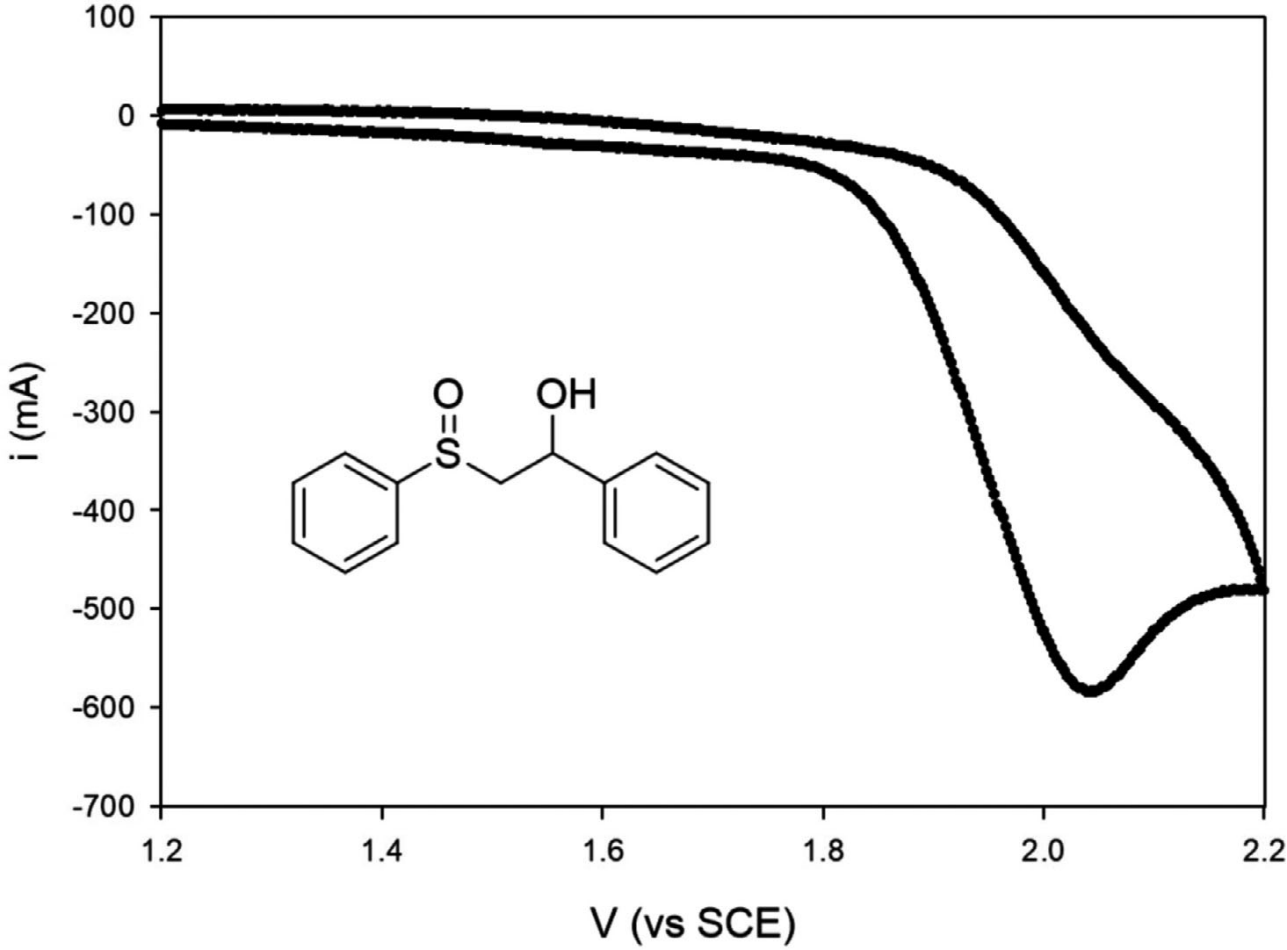
Cyclic voltammogram for **1** in MeCN

Sulfoxides **1**–**6** were electrochemically characterized by cyclic voltammetry experiments. The experiments were carried out on a solution of **1**–**6** (2 mm) in dry MeCN under inert atmosphere at 25 °C, using Bu_4_NBF_4_ (0.1 m) as support electrolyte, an Ag/AgCl (KCl 3 m) reference electrode with a 0.5 V s^−1^ scan rate. For all the sulfoxides investigated an irreversible oxidation process was observed even at higher scan rates (up to 5 V s^−1^) as described in Fig. 1 for **3** (see Figures S1–S5 for the voltammograms of **2**–**6**). Because of the irreversibility observed, only the anodic peak potential values ($E_{p}^{a}$) were determined, and their values (vs SCE) are reported in Table 1.

**Table 1:** Anodic peak potentials for **1**‐**6** ^†^ and rate constants (*k*_q_) for the fluorescence quenching of ^1^[3‐CN‐NMQ^+^]* by **1**‐**5**.^‡^

Sulfoxide	$E_{p}^{a}$ (V) *vs* SCE	*k*_q_ (× 10^10^ m ^−1^ s^−1^)
**1**	2.04	1.35
**2**	1.89	1.69
**3**	1.85	1.32
**4**	1.77	0.52
**5**	1.84	0.73
**6**	1.80	

† In MeCN at 25 °C using an Ag/AgCl (KCl 3 m) as the reference electrode.

‡ In MeCN at 25 °C.

The measured $E_{p}^{a}$ values span from 1.77 to 2.04 V for **4** and **1,** respectively, with the latter very similar to that measured for methyl phenyl sulfoxide under the same experimental conditions ($E_{p}^{a}$ = 2.01 V *vs* SCE) (21). The differences of $E_{p}^{a}$ values for the sulfoxides **1**–**6** are very likely due to the ability of the α‐alkyl group to stabilize the cation radical in which both the charge and the spin density are mainly located on the sulfinyl group (21). Accordingly in **1,** the primary alkyl group should have a stabilizing effect closer to that observed for the methyl group in methyl phenyl sulfoxide whereas for **2–6** the higher degree of substitution should better stabilize the cation radical thus decreasing the oxidation potential. Comparing the $E_{p}^{a}$ values of **2**–**3** with those of the corresponding methyl ethers (**5** and **6,** respectively), it appears that in both cases the substitution of the hydroxy with a methoxy group is reflected in a slight decrease of the oxidation potential ($\Delta E_{p}^{a}$ = 0.05 V). This is probably due to the presence in **2**–**3** of an intramolecular hydrogen bonding between the hydroxy group and the sulfinyl oxygen atom which determines a decrease of the electron density on the sulfinyl group thus increasing the oxidation potential. In all cases, the $E_{p}^{a}$ values reported in Table 1 are largely below the ^1^[3‐CN‐NMQ^+^]* reduction potential (2.72 V vs. SCE in MeCN) (37) thus allowing an exergonic ET process with all the examined sulfoxides. Accordingly, by applying the Weller equation (50), it results that for all the sulfoxides Δ*G*_ET_ < −16 kcal mol^−1^.

### Fluorescence quenching experiments

Having established the thermodynamic feasibility of the ET process from sulfoxides **1**–**6** to ^1^[3‐CN‐NMQ^+^]*, fluorescence quenching experiments were performed in order to determine the rate constants of the interaction of ^1^[3‐CN‐NMQ^+^]* with the substrates investigated. The measurements were carried out irradiating a deaerated MeCN solution of 3‐CN‐NMQ^+^ (1.7 × 10^−5^ m) at its absorption maximum wavelength (*λ*_max_ = 330 nm) and following the decrease of the emission at its maximum intensity (*λ*_max_ = 425 nm) on increasing the sulfoxide concentration (from 2 × 10^−4^ to 0.01 m). According to the Stern‐Volmer equation, by plotting the (*I*_0_/*I*)‐1 *vs* the sulfoxide concentration, very good linear plots were obtained (Figures S6–S10) from whose slopes the fluorescence quenching kinetic constants (*k*_q_) were obtained (Table 1). For all the sulfoxides examined the rate constant for the ^1^[3‐CN‐NMQ^+^]* quenching is close to the diffusion limit in MeCN (1.9 × 10^10^ m ^−1^ s^−1^) (51) in agreement with a photoinduced ET process.

### Steady‐state photolysis: product study

Steady‐state photolysis experiments were carried out irradiating an Ar‐saturated MeCN solution (5 mL) of the sulfoxide (1 × 10^−2^ m) and 3‐CN‐NMQ^+^ ClO_4_^−^ (2 × 10^−3^ m) at 25 °C in a photoreactor equipped with four fluorescence lamps with a maximum emission at 360 nm. At this wavelength, only the photosensitizer is excited as confirmed by the total absence of oxidation products observed in blank experiments where substrate solutions were irradiated in the absence of the photosensitizer. All the reaction products were identified and characterized by ^1^H NMR and GC‐MS analysis (comparison with authentic specimens and literature data). Product quantitative analysis was performed by ^1^H NMR and GC (products derived from alkyl fragments) and HPLC (sulfur‐containing products). In all cases, the overall material recovery was satisfactory (> 90%).

After irradiation of the β‐hydroxysulfoxide **1**, product analysis did not show the presence of any reaction product even after prolonging the irradiation time to two hours whereas irradiation of **2** led, after only 10 min, to significant amounts of fragmentation reaction products, that is, benzaldehyde, benzyl methyl ketone, diphenyl disulfide and phenyl phenylthiosulfonate (entries 1 and 2 in Table 2). On increasing the irradiation time to 30 min the same reaction products were observed with higher yields but they were accompanied by other unknown products probably derived from overoxidation. In Table 2, products and yields for the photosensitized oxidation of the corresponding methyl ether **5** under the same reaction condition are also reported (entries 3 and 4). The products observed are the same as for **2** with the additional formation of 1‐phenyl‐2‐methoxy‐1‐propanol and, after the same irradiation time (10 min), the overall no sulfur‐containing product yields for **5** (3.4%) is slightly higher than that for **2** (2.6%).

**Table 2:** Products and yields for the 3‐CN‐NMQ^+^ photosensitized oxidation of sulfoxides **1‐6** in MeCN at 25 °C.

Entry	Compound	t (min)	Products and yields (%)^†^				
			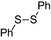	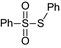	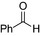	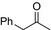	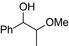
1	**2**	10	0.4	1.3	0.5	2.1	
2	**2**	30	1.2	4.3	2.2	10	
3	**5**	10	0.5	1.6	0.8	0.6	2.0
4	**5**	30	1.6	4.8	1.9	2.0	9.2
			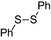	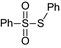	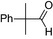		
5	**3**	10	0.6	5.4	11		
6	**3**	30	2.5	6.6	18		
7	**6**	10	0.8	5.9	13		
8	**6**	30	3.1	7.8	21		
			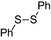	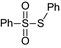	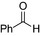	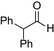	
9	**4**	10	1.4	4.7	13	6.8	

† Yields, average of at least two independent determinations, are referred to the initial amount of substrate.

When the β‐hydroxysulfoxide **3** was irradiated under the same reaction condition of **2**, beside the same sulfur‐containing fragmentation products observed for **2**, 2‐phenyl‐2‐methylpropanal was the exclusive product deriving from the alkyl moiety (entries 5 and 6 in Table 2). As already observed for **2**, also for **3** a prolongation of the irradiation time to 30 min resulted in the formation of the same reaction products in higher yields but accompanied by unidentified products. A comparison with the results obtained with **2** shows a significantly increased product yield for the photosensitized oxidation of **3** with the overall yield, for the fragmentation products deriving from the alkyl moiety, passing from 2.6% to 11% after 10 min irradiation for **2** and **3,** respectively. The photooxidation of the corresponding methyl ether **6** afforded the same fragmentation products observed for **3** but with slightly higher yields (entries 7 and 8 in Table 2).

A 10 min irradiation of the β‐hydroxysulfoxide **4** under the same reaction condition as for **2** and **3**, leads to the formation of the fragmentation products (entry 9 in Table 2): benzaldehyde and diphenylacetaldehyde from the alkyl moiety, diphenyl disulfide and phenyl phenylthiosulfonate from the sulfur‐containing one. Comparison between entry 9 with entries 1 and 5 in Table 2, shows that the photooxidation of **4** is more efficient than those of **2** and **3** with an overall yield for products deriving from the alkyl moiety of 20%.

### Laser flash photolysis study

**Figure 2:**
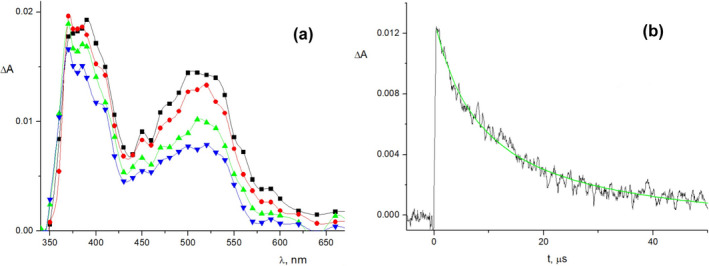
(a) Time‐resolved absorption spectra obtained in the photolysis of sulfoxide **3** (3.0 × 10^−3^ m) in the presence of 3‐CN‐NMQ^+^ ClO_4_^−^ (0.5 × 10^−4^ m) and toluene (1 m) in N_2_‐saturated MeCN at 25 °C registered at 0.18 (■), 1.1 (●), 3.6 (▲) and 6.4 μs (▼) after laser pulse (*λ*_ecc_ = 355 nm). (b) **3^+•^** absorbance decay at 520 nm; the full line represents the 1^st^ order best fitting of the experimental data.

To have a deeper insight in both the nature and the reactivity of the reaction intermediates involved in these processes, a laser flash photolysis (LFP) investigation was carried out. LFP experiments were carried out in N_2_‐saturated MeCN at 22 °C in the presence of 1 m toluene as cosensitizer to minimize the BET process and thus increasing the cation radical yield (52). For all the substrates investigated, the time‐resolved absorption spectra show the formation of two transient species absorbing at ca 390 and 520 nm, respectively (Fig. 2, for **3** and Figures S11–S15 for **1**, **2**, **4**, **5,** and **6**). On the basis of the absorption spectra of aromatic sulfoxide cation radicals reported in the literature (21, 24), the absorption band at 520 nm can be assigned to **1^+•^**‐**6^+•^** whereas the absorption band at 390 nm can be assigned to the 3‐CN‐NMQ**^•^** radical (24).

The observed intermediates confirm the occurrence of an ET process, from the sulfoxides to the singlet excited state of 3‐CN‐NMQ^+^, as the main reaction process (Scheme 2). Interestingly, the absorbance decay at 520 nm does not follow the same kinetic order for all of the substrates. A clean 1^st^ order kinetic profile is only observed for **3^+•^**, **4^+•^** and **6^+•^** (Fig. 2 for **3** and Figures S13 and S15 for **4** and **6**) whereas the other cation radicals show a 2^nd^ order (**1^+•^**) or a mixed 1^st^–2^nd^ order (**2^+•^** and **5^+•^**) kinetic profile (Figures S11, S12 and S14). This observation suggests that only **3^+•^**, **4^+•^** and **6^+•^** mainly undergo unimolecular fragmentation process whereas for the other cation radicals their decay is partially (**2^+•^** and **5^+•^**) or totally (**1^+•^**) associated with a BET process. Such a different behavior is in full agreement with the steady‐state photolysis experiments results (Table 2) where product yields for **3**, **4** and **6** (entries 5, 7 and 9), for which the unproductive BET poorly competes with the fragmentation process, are significantly higher than those for **2** and **5** (entries 1 and 3). Accordingly, **1**, whose cation radical exclusively undergoes BET, was unreactive even at prolonged irradiation times. The values of the 1^st^ order rate constants, *k*_frag_, obtained for **3^+•^**, **4^+•^** and **6^+•^** are reported in Table 3.

**Table 3:** 1^st^ order rate constants for the fragmentation process of cation radicals **3^+•^, 4^+•^** and **6^+•^** in MeCN at 22 °C

Sulfoxide cation radical	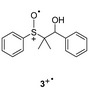	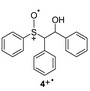	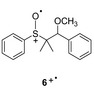
*k*_frag_ (s^−1^)	8.7 × 10^4^	7.0 × 10^5^	2.3 × 10^5^

Comparison between the *k*_frag_ values for **3^+•^** and **6^+•^** shows that the presence of the β‐methoxy group slightly speeds up the cation radical fragmentation process with respect to the β‐hydroxy group. The reason for the lower fragmentation rate observed for **3^+•^** could be tentatively attributed to a slight stabilizing effect related to the presence of an intramolecular hydrogen bond between the β‐hydroxy group and the oxygen‐centered radical as recently reported for the stabilizing effect of alkoxyl radicals by hydrogen bonding (53). Interestingly, the higher fragmentation rate constant observed for **6^+•^** seems to be reflected in the slightly higher product yields observed in the photooxidation of **6** with respect to those for **3** (Compare entries 5 and 7 in Table 2). Indeed, a higher fragmentation rate makes this process more competitive toward the unproductive BET. In the same way, the significantly faster fragmentation process of **4^+•^**, with respect to those of **3^+•^** and **6^+•^**, accounts for the higher product yields observed in the photooxidation of **4** (compare entries 5 and 9 in Table 2).

## DISCUSSION

The results obtained in the 3‐CN‐NMQ^+^ photosensitized oxidation of sulfoxides **2**–**6** show the formation of significant amount of fragmentation products. No products were observed in the oxidation of **1**, a result that can be attributed to the low fragmentation rate for the cation radical **1^+•^** that favors the competitive and unproductive BET process as shown by the LFP experiments. For **1^+•^**, the C_α_‐S bond cleavage rate is expected to be slower than those for the cation radicals **2^+•^‐6^+•^** because of the primary alkyl group bonded to the sulfur atom. Moreover, the presence of the β‐OH group seems not to be able to induce an efficient C_α_‐C_β_ bond fragmentation as observed for the β‐hydroxy sulfides (35).

In a previous study, it was observed that the presence of bases, that is, substituted pyridines, in the reaction medium is reflected in a dramatic acceleration of the C_α_‐C_β_ bond cleavage in β‐hydroxysulfide cation radicals (35). Such acceleration was rationalized invoking a transition state where the C_α_‐C_β_ bond cleavage and the intramolecular ET from this bond to the sulfur atom are coupled with the O‐H bond cleavage induced by the base. On these bases, we carried out some 3‐CN‐NMQ^+^ photosensitized oxidation reactions on **1** in the presence of pyridine (from 0.001 to 0.1 m) or 4‐CN‐pyridine (from 0.02 to 0.1 m) in order to induce the C_α_‐C_β_ bond cleavage but, also under these reaction conditions, no reaction products were observed. The lack of oxidation products observed also in the presence of pyridines may be likely due to the possibility that these species could favorably compete with **1** in the ET process to the excited sensitizer. In contrast, when more oxidizable sulfides are used as substrates, this possibility can be excluded.

**Scheme 3:**
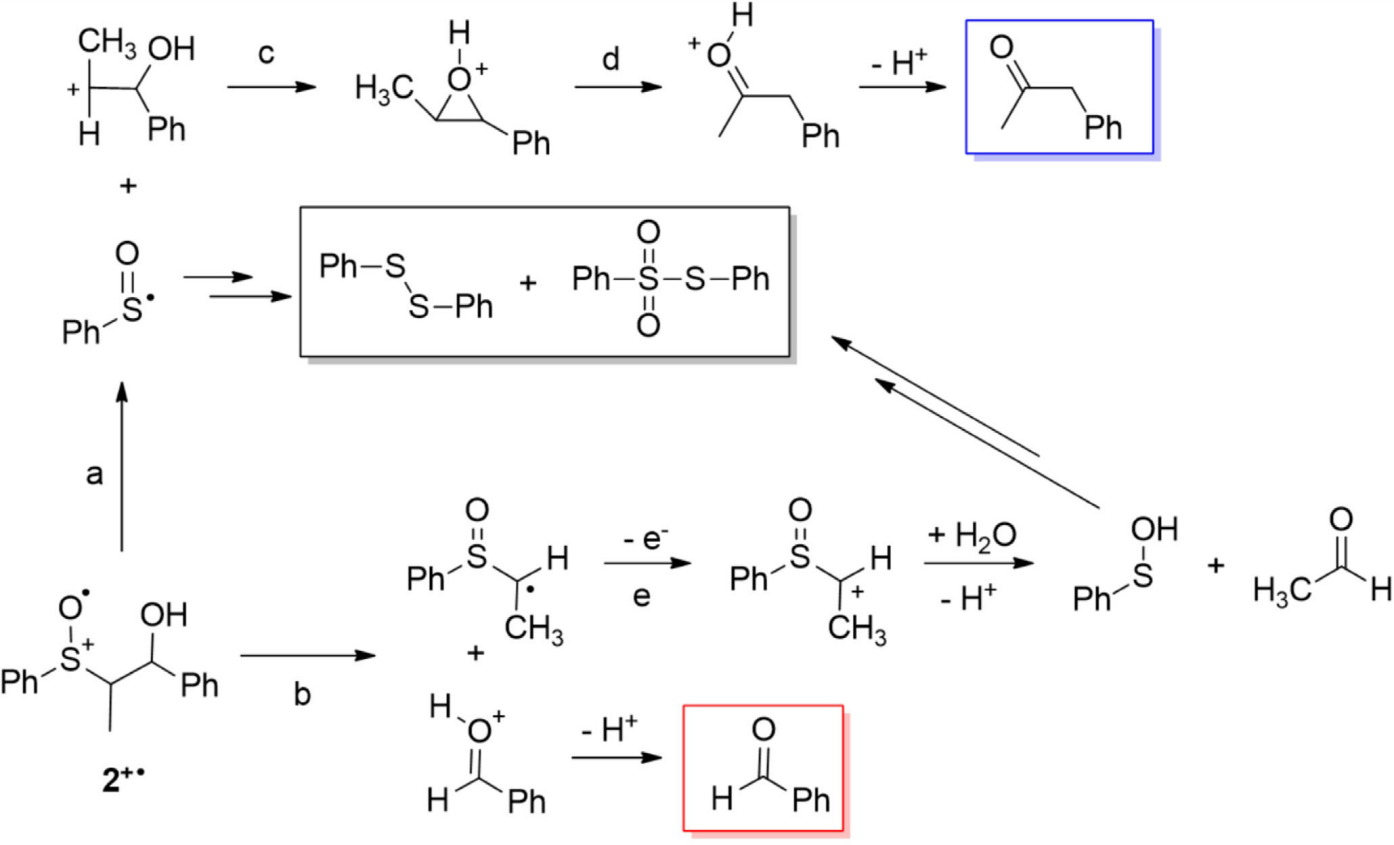
Plausible mechanism for product formation from **2^+•^**. Framed structures represent the observed reaction products deriving from C_α_‐C_β_ (red) and C_α_‐S bond cleavage (blue) or both (black).

In the oxidation of the β‐hydroxysulfoxide **2**, two fragmentation products deriving from the alkyl moiety (benzaldehyde and phenylacetone) are observed accompanied by two sulfur‐containing ones, that is, diphenyl disulfide and phenyl phenylthiosulfonate. The formation of these products suggests that both C_α_‐C_β_ and C_α_‐S bond cleavage take place on **2^+•^** as described in Scheme 3. According to the proposed mechanism, phenylacetone derives from the C_α_‐S bond cleavage (path a) whereas benzaldehyde derives from the C_α_‐C_β_ scission (path b). The mechanism for the formation of phenylacetone from the carbocation C_6_H_5_CH(OH)CH^+^CH_3_ involves the conversion of the latter into a protonated epoxide (path c) followed by a 1,2‐ hydride shift (path d) as already reported (54). Since benzaldehyde and phenylacetone come from competitive C_α_‐C_β_ and C_α_‐S bond cleavage, respectively, from their molar ratio (see entry 1 in Table 2) it is possible to estimate the relative rates for these two processes with the C_α_‐S bond cleavage being ca 4 fold faster than C_α_‐C_β_ one.

**Scheme 4:**
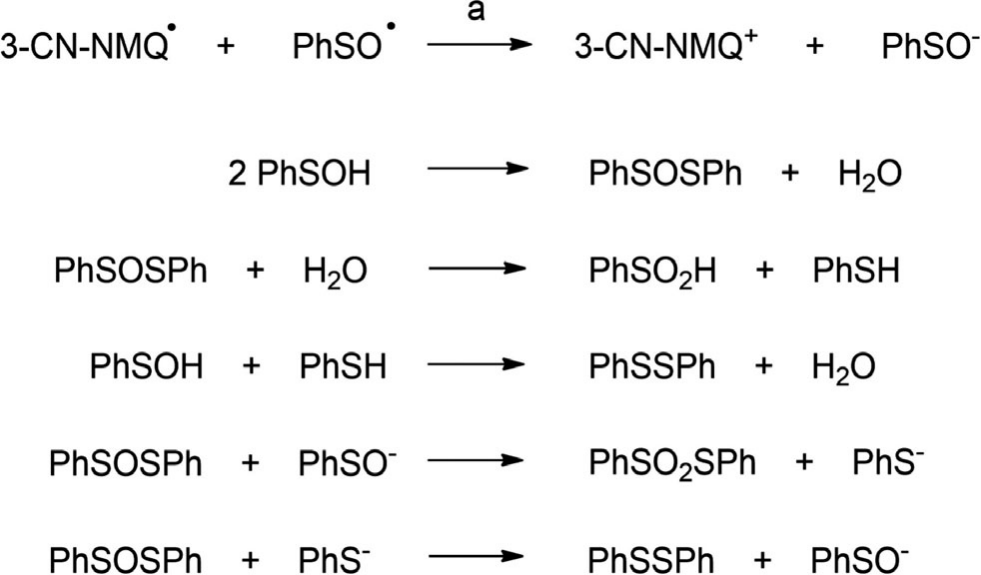
Proposed mechanism for the conversion of the phenyl sulfinyl radical to the sulfur‐containing products observed in the photooxidation of **2**‐**6** sensitized by 3‐CN‐NMQ^+^.

The sulfur‐containing products observed in the photooxidation of **2^+•^** are those expected to derive from the phenyl sulfinyl radical formed upon C_α_‐S bond scission. Accordingly, the same products were already observed in previous studies concerning the photosensitized oxidation of alkyl aryl sulfoxides (24), and their formation was proposed to involve the first reduction of the phenyl sulfinyl radical to phenyl sulfenate by the reduced form of 3‐cyano‐*N*‐methyl‐quinolinium, 3‐CN‐NMQ**^•^** (Scheme 4, path a). This process is thermodynamically favored since the reduction potential of PhSO^•^ (1.08 V *vs* SCE in MeCN) (24) is much higher than that of 3‐CN‐NMQ^+^ (−0.60 V *vs* SCE in MeCN) (55) and accounts for the high photosensitizer recovery observed in the reaction mixtures after irradiation (> 77%). Once formed, the sulfenate anion or its protonated form, sulfenic acid, are converted to the observed products PhSSPh and PhSO_2_SPh as described in Scheme 4 (36).

**Scheme 5:**
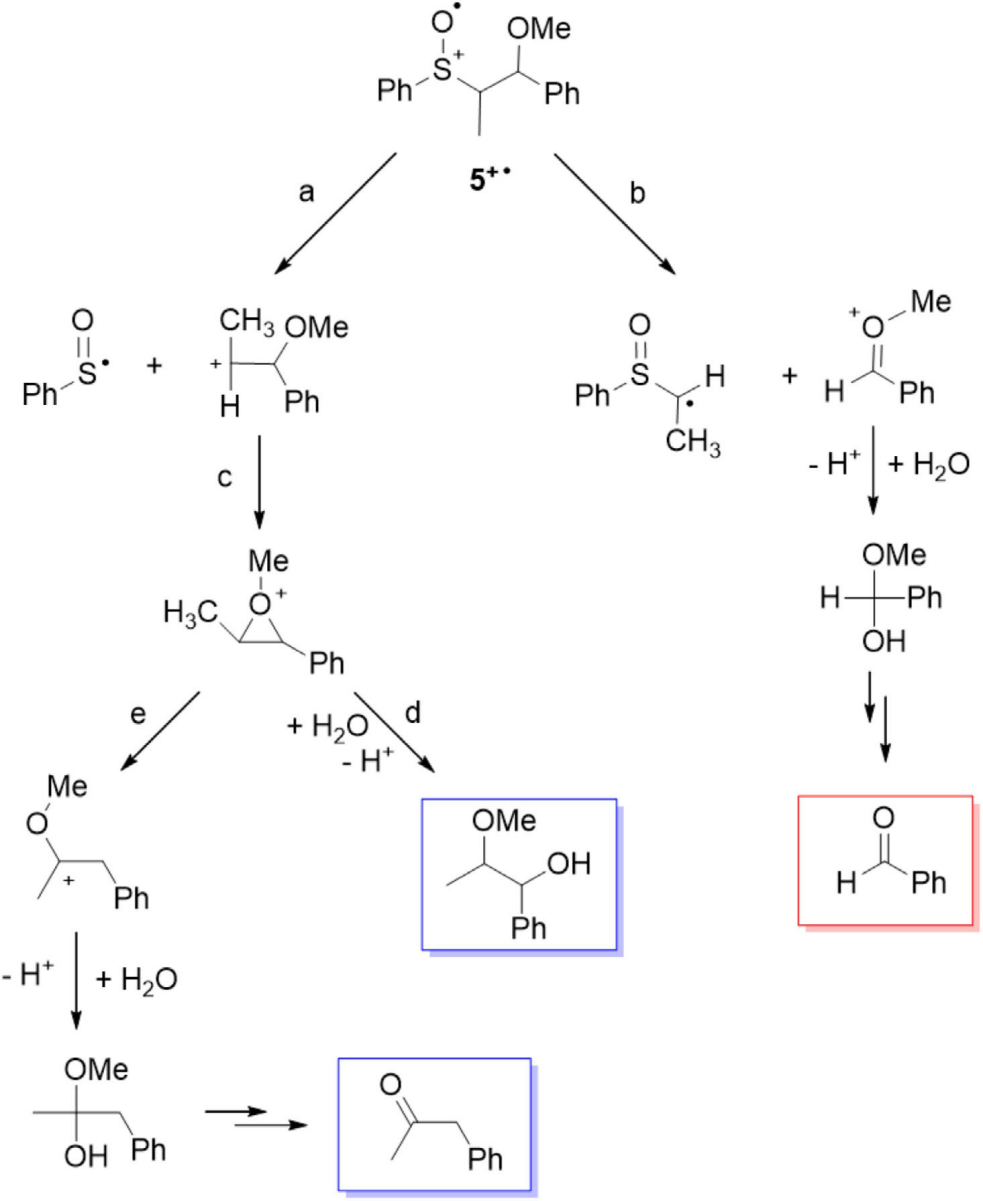
Proposed mechanism for product formation from **5^+•^**. Framed structures represent the observed reaction products deriving from C_α_‐C_β_ (red) and C_α_‐S bond cleavage (blue).

As described in Scheme 3 (path b), the C_α_‐C_β_ bond cleavage on **2^+•^** leads to the formation, besides benzaldehyde, of an α‐sulfinylmethyl radical. Since there was no evidence for the formation of its dimerization product, a possible fate for this species could involve its oxidation to the corresponding carbocation followed by reaction with trace of water to form sulfenic acid (and then PhSSPh and PhSO_2_SPh) and acetaldehyde (Scheme 3, path e). The photoinduced oxidation of the β‐methoxy sulfoxide **5** provides the same reaction products observed for the corresponding β‐hydroxy sulfoxide **2** accompanied by a significant amount of 2‐methoxy‐1‐phenyl‐1‐propanol. According to the mechanism proposed for **2** (Scheme 3), product formation from **5** can be rationalized as described in Scheme 5 where the carbocation deriving from the C_α_‐S bond cleavage (path a) can be converted into an oxiranium intermediate (path c) that can undergo both nucleophilic water addition (path d) to give 2‐methoxy‐1‐phenyl‐1‐propanol or 1,2‐ hydride shift (path e) to provide a carbocation precursor of benzyl methyl ketone. The alkyl fragment deriving from the C_α_‐C_β_ bond cleavage (path b), after water addition, provides the hemiacetal precursor of benzaldehyde. On the basis of the relative amount of 2‐methoxy‐1‐phenyl‐1‐propanol, benzyl methyl ketone and benzaldehyde (Table 2), a significant predominance of C_α_‐S over C_α_‐C_β_ bond cleavage seems to take place within **5^+•^**.

**Scheme 6:**
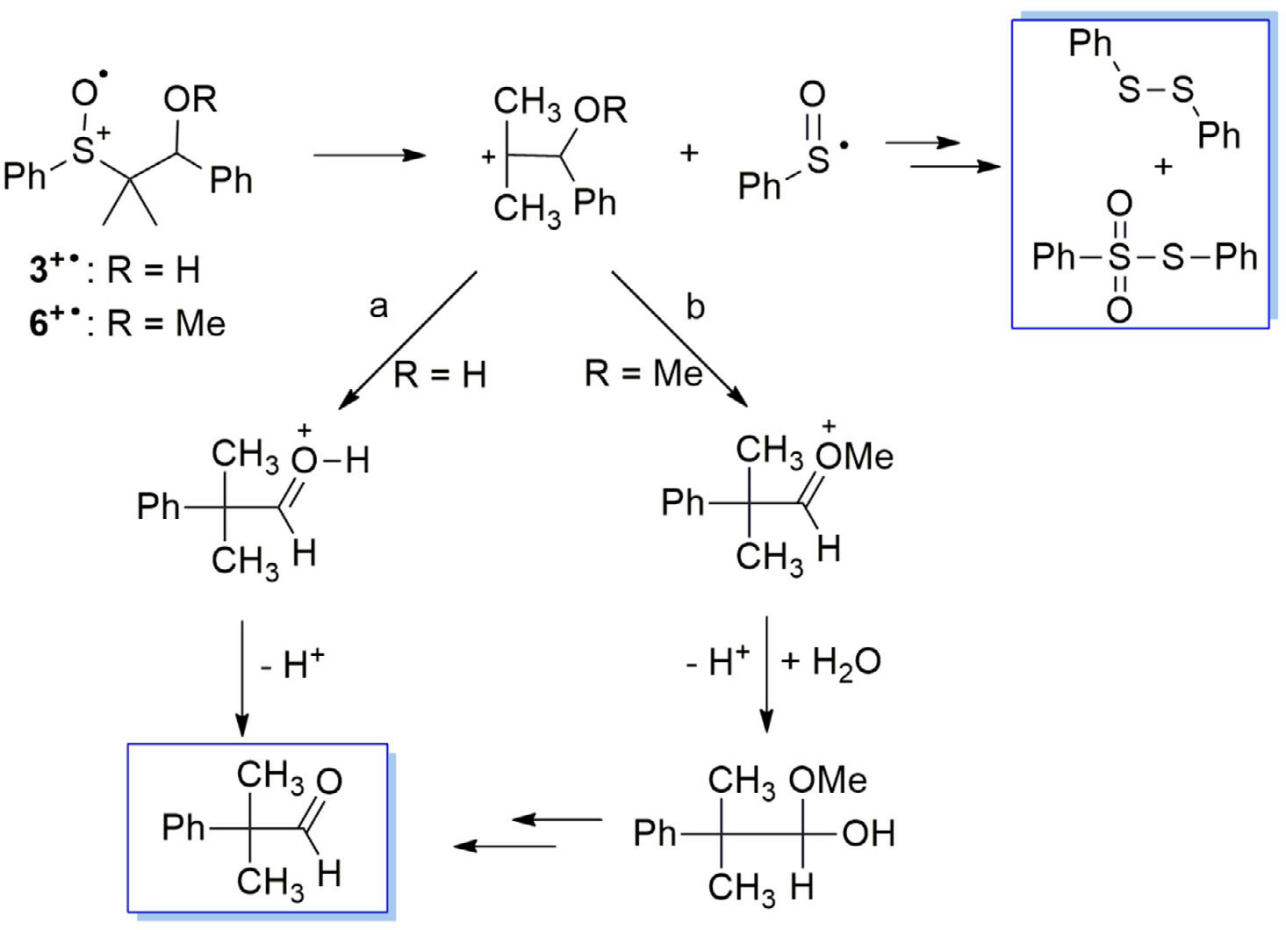
Proposed mechanism for product formation from **3^+•^** and **6^+•^**. Framed structures represent the observed reaction products deriving from C_α_‐S bond cleavage.

The photooxidation of **3** and **6**, afforded 2‐methyl‐2‐phenylpropanal as the exclusive product deriving from the alkyl moiety whereas the sulfur‐containing products were the same as observed for **2**: diphenyl disulfide and phenyl phenythiosulfonate (Table 2, entries 5–8). This result suggests the occurrence of C_α_‐S bond cleavage as the exclusive fragmentation process for **3^+•^** and **6^+•^** (Scheme 6). The formation of 2‐methyl‐2‐phenylpropanal from **3^+•^** and **6^+•^** can be rationalized taking into account a 1,2‐phenyl shift within the carbocation PhCH(OR)C(CH_3_)_2_^+^ (Scheme 6, path a for **3^+•^** and b for **6^+•^**) as already reported (54).

**Scheme 7:**
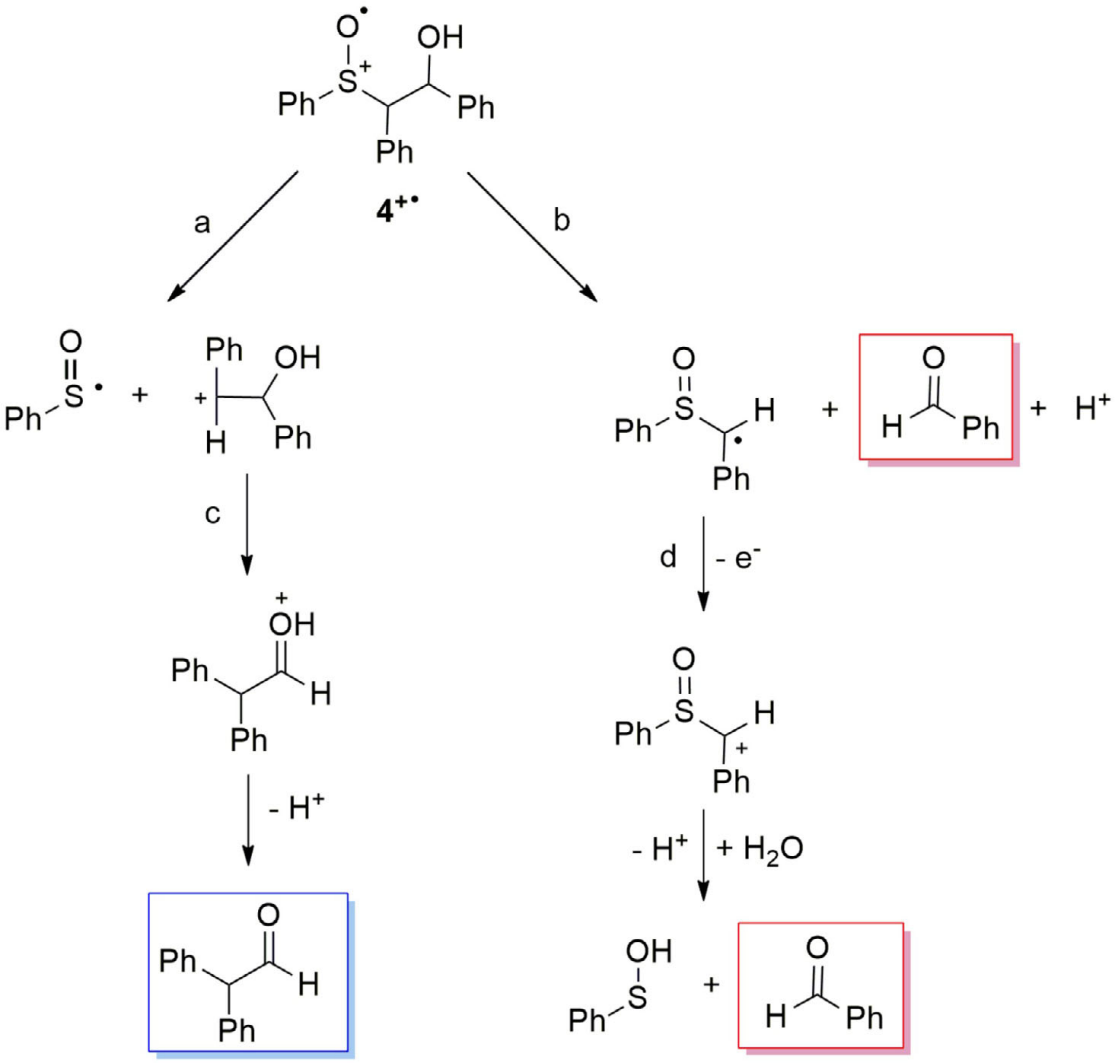
Proposed mechanism for product formation from **4^+•^**. Framed structures represent the observed reaction products deriving from C_α_‐C_β_ (red) and C_α_‐S bond cleavage (blue).

The formation of diphenylacetaldehyde, and benzaldehyde from the alkyl moiety, and diphenyl disulfide and phenyl phenylthiosulfonate as sulfur‐containing products (Table 2, entry 9) in the photooxidation of **4** suggests that **4^+•^** can undergo both C_α_‐S and C_α_‐C_β_ bond cleavage as described in Scheme 7 where diphenylacetaldehyde derives from the C_α_‐S scission (path a) followed by a 1,2‐phenyl shift within the carbocation thus formed (path c) as previously proposed for **3^+•^** (54). In contrast, benzaldehyde is formed upon C_α_‐C_β_ bond cleavage (path b) in a similar way as described for the same process with **2**. Moreover, it should be considered that for **4^+•^** the C_α_‐C_β_ bond cleavage leads not only to benzaldehyde but also to the formation of an α‐sulfinyl radical that, upon further oxidation (path d), should convert to a sulfenic acid and a second benzaldehyde molecule following the pathway proposed above for **2^+•^**.

Under the assumption that two molecules of benzaldehyde are formed upon C_α_‐C_β_ fragmentation, the corrected benzaldehyde and diphenylacetaldehyde yields ratio (Table 2, entry 9) indicates an almost equal percentage of C_α_‐C_β_ and C_α_‐S bond cleavage thus suggesting that the presence of the α‐phenyl group can stabilize to the same extent both the secondary benzylic carbocation, from C_α_‐S scission, and the secondary benzylic α‐sulfinyl radical deriving from the C_α_‐C_β_ scission.

The collected results show that the presence of a β‐hydroxy or ‐methoxy substituent in the sulfoxide cation radicals investigated renders the C_α_‐C_β_ bond cleavage competitive with the C_α_‐S scission. The extent of this competition appears to be highly dependent on the relative stabilities of the fragments formed from these two processes. Since all the β‐hydroxysulfoxide cation radicals afford two common fragments from the C_α_‐S and C_α_‐C_β_ bond cleavage, that is, PhSO**^•^** and [PhCHOH]^+^ respectively, the competition between these two processes is only governed by the relative stability of the other two fragments formed: PhCH(OH)C^+^RR’ from C_α_‐S fragmentation and PhS(O)C**^•^** RR’ from C_α_‐C_β_ bond cleavage. Thus, the exclusive C_α_‐S bond cleavage observed for **3^+•^** should be likely due to the stability of the tertiary carbocation formed that would result higher than that of the alkyl radical deriving from the C_α_‐C_β_ scission. Moreover, the observation that the C_α_‐C_β_ bond cleavage competes more favorably in **4^+•^** (C_α_‐S/C_α_‐C_β_ ≈ 1) rather than in **2^+•^** (C_α_‐S/C_α_‐C_β_ ≈ 4) suggests that the α‐phenyl substituent would stabilize the radical fragment formed upon C_α_‐C_β_ cleavage (PhS(O)C**^•^** HPh), with respect to the carbocation deriving from the C_α_‐S scission (PhCH(OH)CH^+^Ph), more efficiently than the α‐methyl group. Accordingly, the lower stabilities of both the primary carbocation and radical that would have been formed from the C_α_‐S and C_α_‐C_β_ bond cleavage in **1^+•^** determine a fragmentation rate too low to compete with the unproductive BET.

Concerning the role of the hydroxyl substituent on the cation radical fragmentation, both product analysis and LPF experiments on **2**–**5** and **3**–**6** show a negligible effect of the substitution of the ‐OH with the ‐OMe group. These observations suggest that the previously proposed favorable effect on the C_α_‐C_β_ bond cleavage, within β‐hydroxysulfide cation radicals, exerted by a hydrogen bonding between the ‐OH and the solvent in the transition state (35) is no longer valid for β‐hydroxysulfoxides. Thus, the favorable effect of the β‐oxygenated substituents on the C_α_‐C_β_ bond scission seems to be exclusively due to the stabilization of the formed carbocation, exerted by the vicinal oxygen atom.

To better investigate the role of a β‐oxygenated group (‐OH or ‐OMe) on the C_α_‐S bond cleavage within a sulfoxide cation radical, it could be worthy of interest to compare the *k*_frag_ values for **3^+•^** and **6^+•^** with that of the *t*‐butyl phenyl sulfoxide cation radical previously measured under the same experimental condition (24). As for **3^+•^** and **6^+•^**, the cation radical of *t*‐butyl phenyl sulfoxide has a tertiary alkyl group bonded to the sulfur atom and was shown to exclusively undergo C_α_‐S bond cleavage with a rate constant of 1.6 × 10^6^ s^−1^, one order of magnitude higher than those measured for **3^+•^** and **6^+•^** (Table 3). Interestingly, the presence of the β‐oxygenated substituent seems to stabilize the cation radical thus discarding a significant contribution of an intramolecular nucleophilic assistance, exerted by the β‐OH or ‐OMe substituent, on the C_α_‐S bond cleavage. Such a stabilization could be associated with the formation of a two center‐three electrons intramolecular bond between the β‐oxygen atom and the cation radical localized on the sulfinyl group, as previously proposed for the intermolecular stabilization of DMSO cation radical by water molecules (56).

## CONCLUSION

All the β‐hydroxysulfoxides investigated in this work have been shown to rapidly react with the singlet excited state of 3‐CN‐NMQ^+^ via an ET process affording the corresponding cation radicals as unequivocally revealed by LFP experiments. Once formed, the cation radicals can undergo either BET or fragmentation processes whose competition depends on the relative stability of the fragments formed in the latter process. Beside the expected C_α_‐S bond cleavage, product analysis in steady‐state photolysis experiments showed, in some cases, the occurrence of the unprecedented C_α_‐C_β_ bond scission as well. In this case too, the competition between these two fragmentation processes in β‐hydroxysulfoxides cation radicals seems to depend mainly on the relative stability of the fragments formed. Finally, the similar behavior observed for the β‐methoxysulfoxides **5** and **6** and the corresponding β‐hydroxysulfoxides (**2** and **3**) in both LFP and steady‐state experiments clearly indicates that the role of the β‐hydroxy substituent in promoting the C_α_‐C_β_ bond cleavage is limited to the stabilization of the α‐carbocation formed. This evidence discards the hypothesis of a solvent assistance, via hydrogen bonding, previously proposed for the same process in β‐hydroxysulfides cation radicals.

## Supporting information


**Figure S1**. Cyclic voltammogram of **2** in MeCN.
**Figure S2**. Cyclic voltammogram of **3** in MeCN.
**Figure S3**. Cyclic voltammogram of **4** in MeCN.
**Figure S4**. Cyclic voltammogram of **5** in MeCN.
**Figure S5**. Cyclic voltammogram of **6** in MeCN.
**Figure S6**. Stern‐Volmer plot for the fluorescence quenching of ^1^[3‐CN‐NMQ^+^]* by **1** at different concentration in MeCN at 25 °C.
**Figure S7**. Stern‐Volmer plot for the fluorescence quenching of ^1^[3‐CN‐NMQ^+^]* by **2** at different concentration in MeCN at 25 °C.
**Figure S8**. Stern‐Volmer plot for the fluorescence quenching of ^1^[3‐CN‐NMQ^+^]* by **3** at different concentration in MeCN at 25 °C.
**Figure S9**. Stern‐Volmer plot for the fluorescence quenching of ^1^[3‐CN‐NMQ^+^]* by **4** at different concentration in MeCN at 25 °C.
**Figure S10**. Stern‐Volmer plot for the fluorescence quenching of ^1^[3‐CN‐NMQ^+^]* by **5** at different concentration in MeCN at 25 °C.
**Figure S11**. LFP experiment for **1**.
**Figure S12**. LFP experiment for **2**.
**Figure S13**. LFP experiment for **4**.
**Figure S14**. LFP experiment for **5**.
**Figure S15**. LFP experiment for **6**.
**Figure S16**. ^1^H NMR spectrum of **5** in CDCl_3_.
**Figure S17**. ^13^C NMR spectrum of **5** in CDCl_3_.
**Figure S18**. ^1^H NMR spectrum of **6** in CDCl_3_.
**Figure S19**. ^13^C NMR spectrum of **6** in CDCl_3_.
**Figure S20**. FT‐IR spectrum of **5**.
**Figure S21**. FT‐IR spectrum of **6**.
