# Supplementary material for: Competition Between Cα‐S and Cα‐Cβ Bond Cleavage in β‐Hydroxysulfoxides Cation Radicals Generated by Photoinduced Electron Transfer
Source: Photochem Photobiol. 2021 Jun 3;97(6):1310–21. doi: 10.1111/php.13455 (PMC9290654; doi:10.1111/php.13455)
Supplement: Supplementary file 1 — Figure S1. Cyclic voltammogram of 2 in MeCN. Figure S2. Cyclic voltammogram of 3 in MeCN. Figure S3. Cyclic voltammogram of 4 in MeCN. Figure S4. Cyclic voltammogram of 5 in MeCN. Figure S5. Cyclic voltammogram of 6 in MeCN. Figure S6. Stern‐Volmer plot for the fluorescence quenching of 1[3‐CN‐NMQ+]* by 1 at different concentration in MeCN at 25 °C. Figure S7. Stern‐Volmer plot for the fluorescence quenching of 1[3‐CN‐NMQ+]* by 2 at different concentration in MeCN at 25 °C. Figure S8. Stern‐Volmer plot for the fluorescence quenching of 1[3‐CN‐NMQ+]* by 3 at different concentration in MeCN at 25 °C. Figure S9. Stern‐Volmer plot for the fluorescence quenching of 1[3‐CN‐NMQ+]* by 4 at different concentration in MeCN at 25 °C. Figure S10. Stern‐Volmer plot for the fluorescence quenching of 1[3‐CN‐NMQ+]* by 5 at different concentration in MeCN at 25 °C. Figure S11. LFP experiment for 1. Figure S12. LFP experiment for 2. Figure S13. LFP experiment for 4. Figure S14. LFP experiment for 5. Figure S15. LFP experiment for 6. Figure S16. 1H NMR spectrum of 5 in CDCl3. Figure S17. 13C NMR spectrum of 5 in CDCl3. Figure S18. 1H NMR spectrum of 6 in CDCl3. Figure S19. 13C NMR spectrum of 6 in CDCl3. Figure S20. FT‐IR spectrum of 5. Figure S21. FT‐IR spectrum of 6. [file PHP-97-1310-s001.pdf]

Supporting Information

Competition Between C_α-S and C_α-C_β Bond Cleavage in β-Hydroxysulfoxides Cation Radicals Generated by Photoinduced Electron Transfer.

Andrea Lapi*, Claudio D'Alfonso, Tiziana Del Giacco, Osvaldo Lanzalunga

*Corresponding author e-mail: andrea.lapi@uniroma1.it (Andrea Lapi)

Table of contents

**Figure S1.**	Cyclic voltammogram of **2** in MeCN.	Pag. 3
**Figure S2.**	Cyclic voltammogram of **3** in MeCN.	Pag. 3
**Figure S3.**	Cyclic voltammogram of **4** in MeCN.	Pag. 4
**Figure S4.**	Cyclic voltammogram of **5** in MeCN.	Pag. 4
**Figure S5.**	Cyclic voltammogram of **6** in MeCN.	Pag. 5
**Figure S6.**	Stern-Volmer plot for the fluorescence quenching of ^{1}[3-CN-NMQ$^+$]* by **1**.	Pag. 5
**Figure S7.**	Stern-Volmer plot for the fluorescence quenching of ^{1}[3-CN-NMQ$^+$]* by **2**.	Pag. 6
**Figure S8.**	Stern-Volmer plot for the fluorescence quenching of ^{1}[3-CN-NMQ$^+$]* by **3**.	Pag. 6
**Figure S9.**	Stern-Volmer plot for the fluorescence quenching of ^{1}[3-CN-NMQ$^+$]* by **4**.	Pag. 7
**Figure S10.**	Stern-Volmer plot for the fluorescence quenching of ^{1}[3-CN-NMQ$^+$]* by **5**.	Pag. 7
**Figure S11.**	LFP experiment for **1**.	Pag. 8
**Figure S12.**	LFP experiment for **2**.	Pag. 8
**Figure S13.**	LFP experiment for **4**.	Pag. 9
**Figure S14.**	LFP experiment for **5**.	Pag. 9
**Figure S15.**	LFP experiment for **6**.	Pag. 10
**Figure S16.**	^{1}H NMR spectrum of **5** in $CDCl_3$.	Pag. 11
**Figure S17.**	^{13}C NMR spectrum of **5** in $CDCl_3$.	Pag. 11
**Figure S18.**	^{1}H NMR spectrum of **6** in $CDCl_3$.	Pag. 12
**Figure S19.**	^{13}C NMR spectrum of **6** in $CDCl_3$.	Pag. 12
**Figure S20.**	FT-IR spectrum of **5**.	Pag. 13
**Figure S21.**	FT-IR spectrum of **6**.	Pag. 13

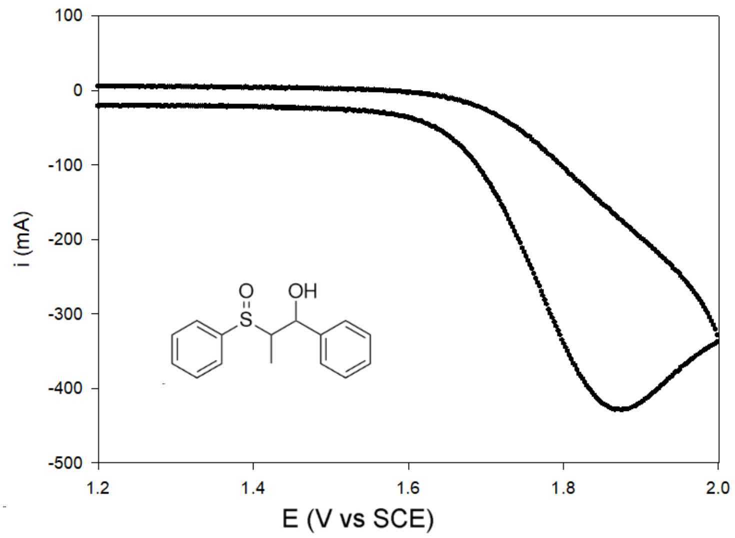

Figure S1. Cyclic voltammogram of **2** in MeCN.

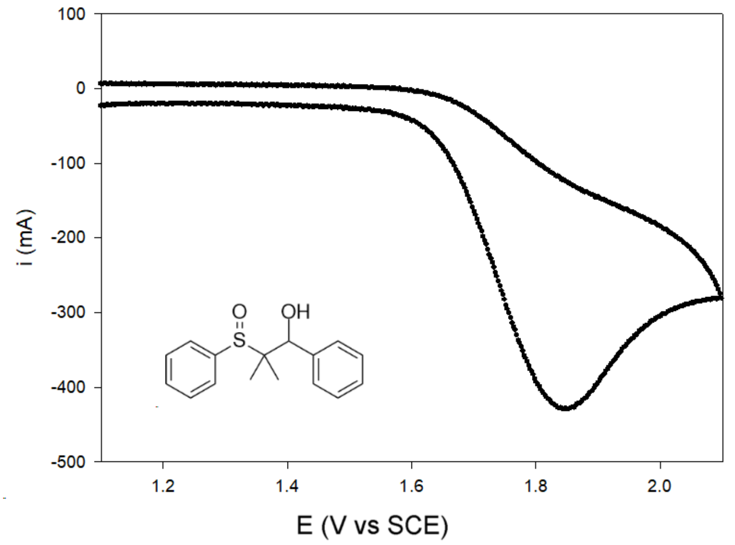

Figure S2. Cyclic voltammogram of **3** in MeCN.

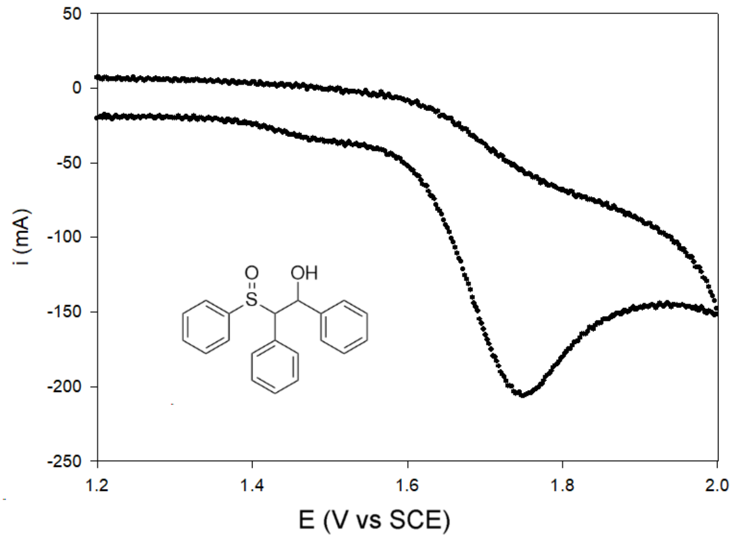

Figure S3. Cyclic voltammogram of **4** in MeCN.

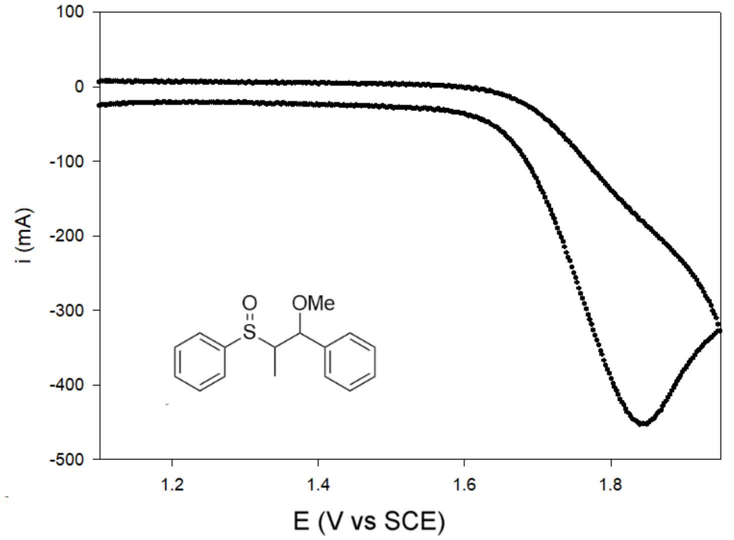

Figure S4. Cyclic voltammogram of **5** in MeCN.

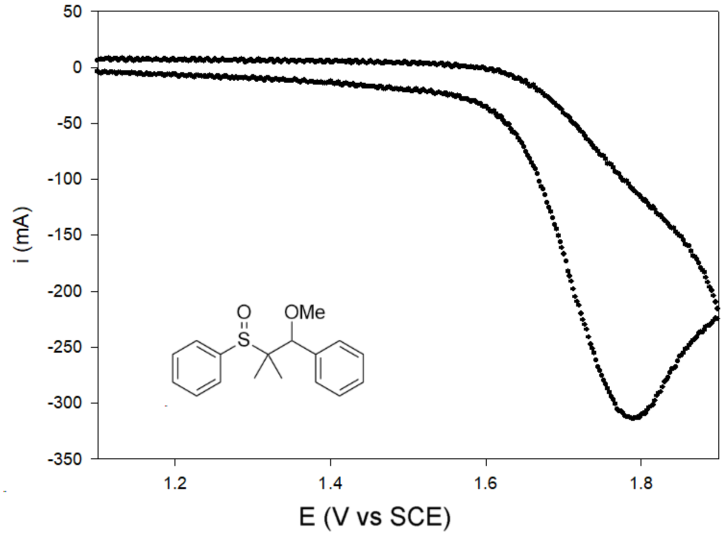

Figure S5. Cyclic voltammogram of **6** in MeCN.

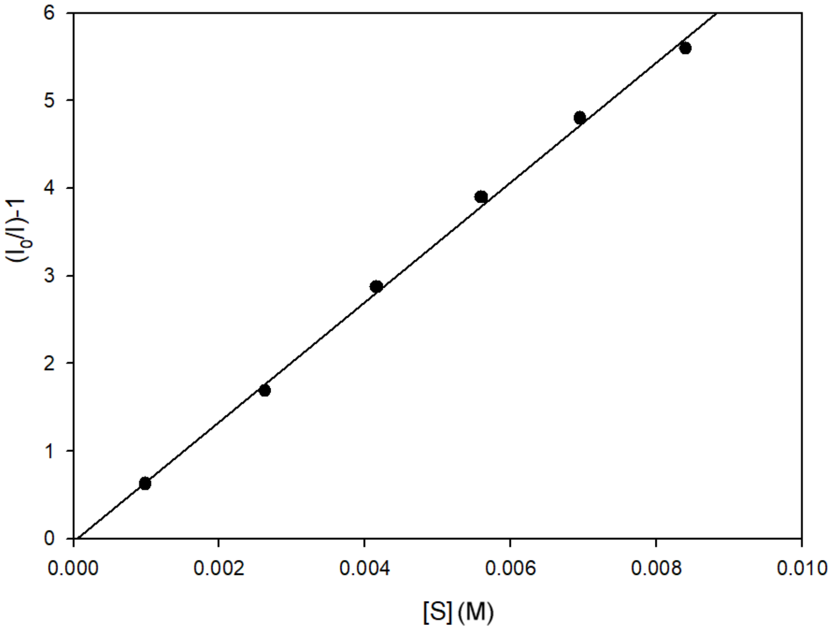

Figure S6. Stern-Volmer plot for the fluorescence quenching of $^{1}[3\text{-CN-NMQ}^{+}]^{*}$ by **1** at different concentration in MeCN at 25 °C.

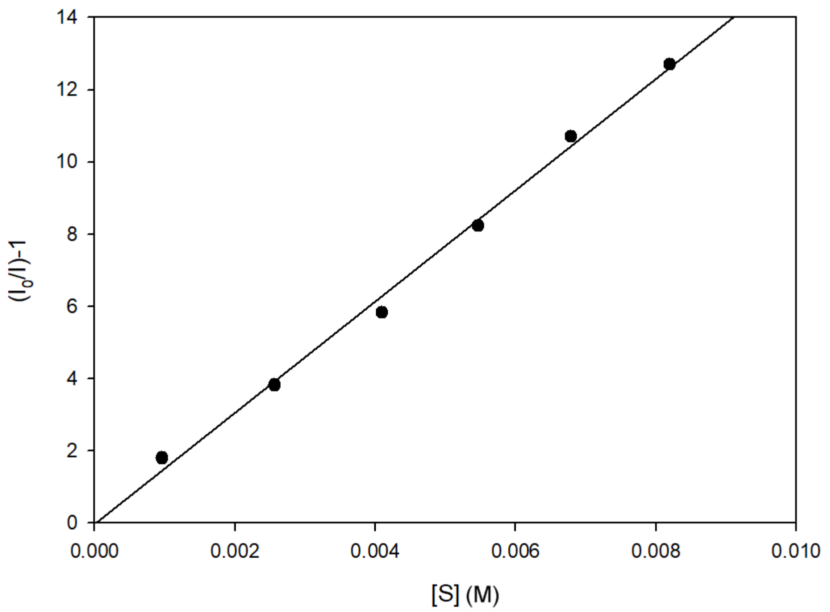

Figure S7. Stern-Volmer plot for the fluorescence quenching of ^{1}[3-CN-NMQ$^+$]* by **2** at different concentration in MeCN at 25 °C.

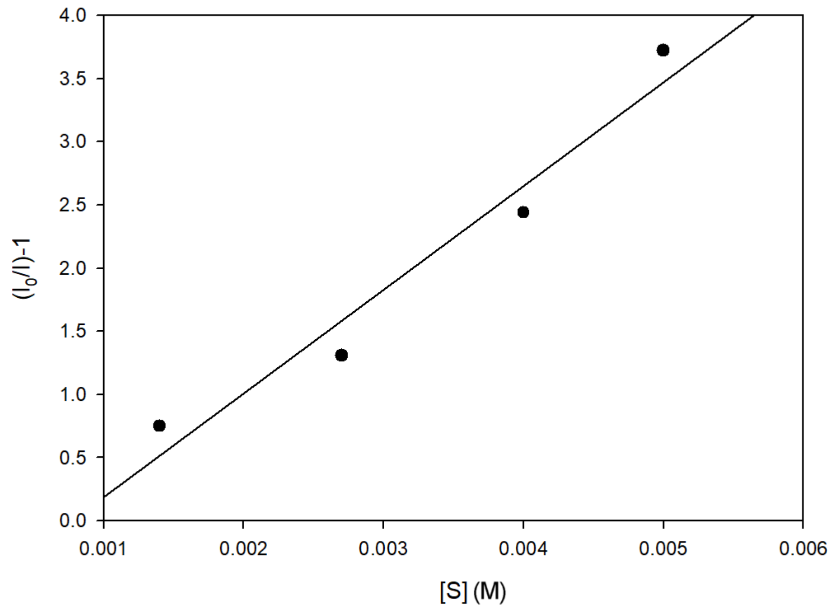

Figure S8. Stern-Volmer plot for the fluorescence quenching of ^{1}[3-CN-NMQ$^+$]* by **3** at different concentration in MeCN at 25 °C.

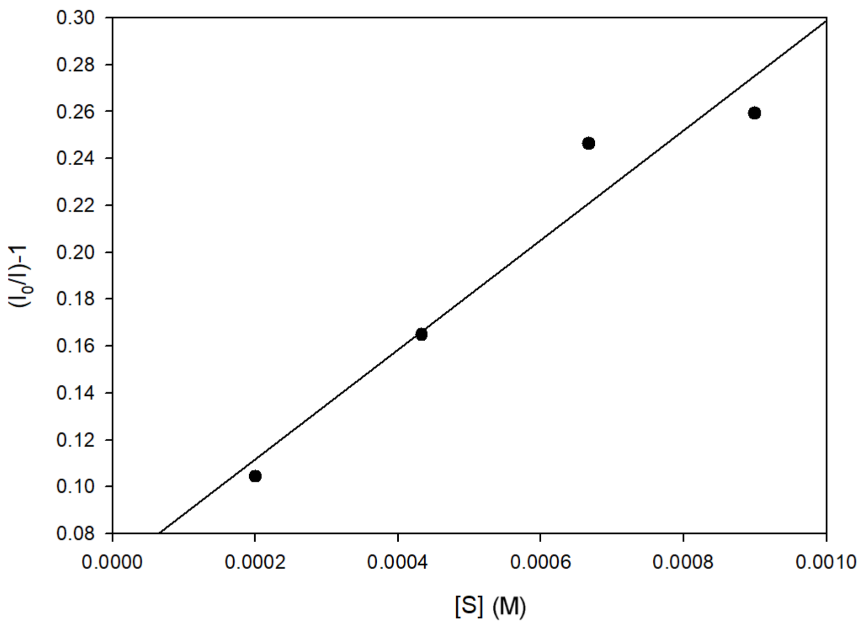

Figure S9. Stern-Volmer plot for the fluorescence quenching of ^{1}[3-CN-NMQ$^+$]* by **4** at different concentration in MeCN at 25 °C.

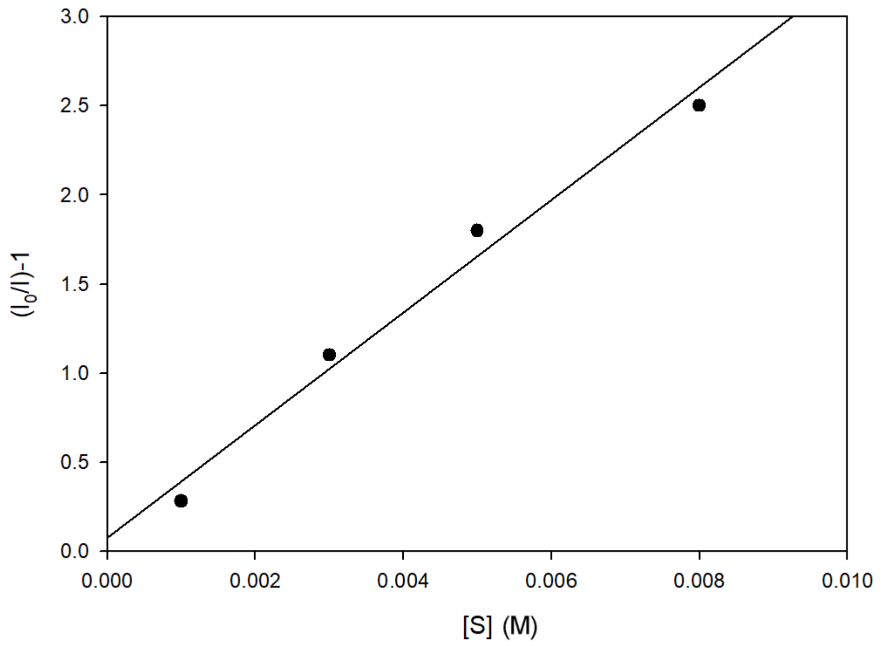

Figure S10. Stern-Volmer plot for the fluorescence quenching of ^{1}[3-CN-NMQ$^+$]* by **5** at different concentration in MeCN at 25 °C.

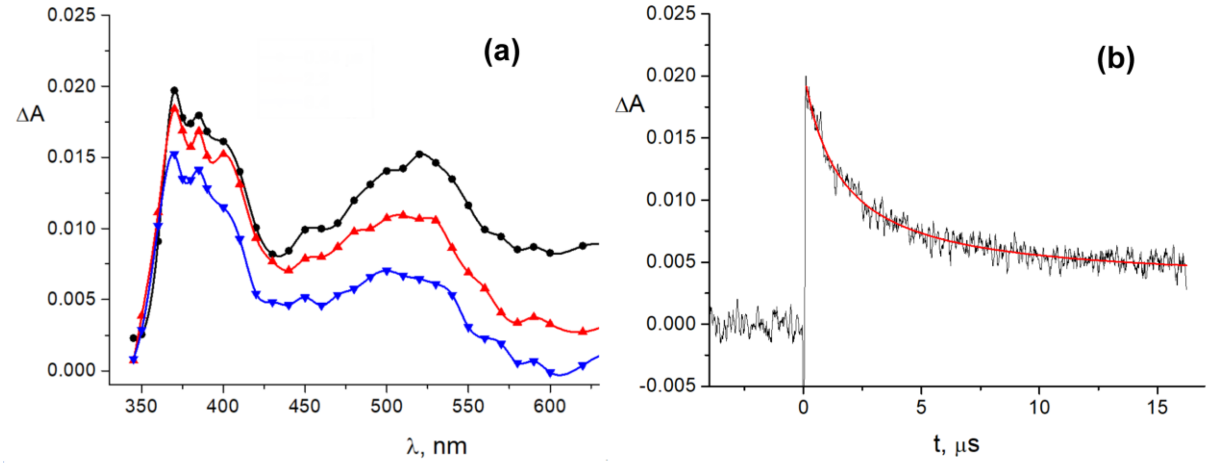

Figure S11. (a) Time resolved absorption spectra obtained in the photolysis of sulfoxide **1** (3.0×10^{-3} M) in the presence of 3-CN-NMQ$^+$ ClO_4^- (0.5×10^{-4} M) and toluene (1 M) in N_2-saturated MeCN at 22 °C registered at 0.94 (■), 2.2 (▲) and 6.4 μs (▼) after laser pulse (λ_{ecc}= 355 nm). (b) Decay kinetic of **1**$^{+\bullet}$ recorded at 510 nm after the laser pulse (λ_{ecc}= 355 nm) in N_2-saturated MeCN; the full line represents the 2nd order best fitting of the experimental data.

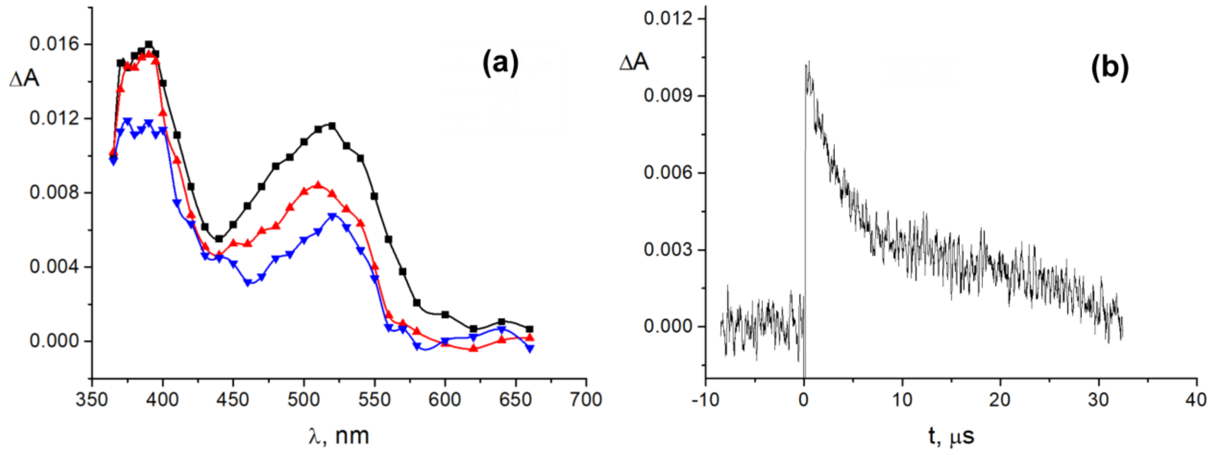

Figure S12. Up: time resolved absorption spectra obtained in the photolysis of sulfoxide **2** (3.0×10^{-3} M) in the presence of 3-CN-NMQ$^+$ ClO_4^- (0.5×10^{-4} M) and toluene (1 M) in N_2-saturated MeCN at 22 °C registered at 0.13 (■) 1.8 (▲) and 6.4 μs (▼)after laser pulse (λ_{ecc}= 355 nm). Down: decay kinetic of **2**$^{+\bullet}$ recorded at 520 nm after the laser pulse (λ_{ecc}= 355 nm) in N_2-saturated MeCN.

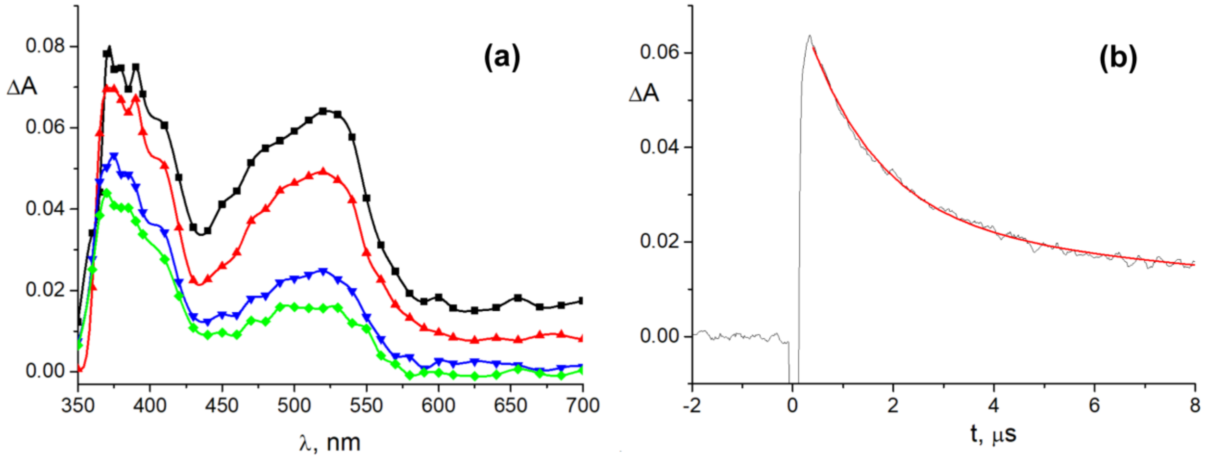

Figure S13. (a) Time resolved absorption spectra obtained in the photolysis of sulfoxide **4** (3.0×10^{-3} M) in the presence of 3-CN-NMQ$^+$ ClO_4^- (0.5×10^{-4} M) and toluene (1 M) in N_2-saturated MeCN at 22 °C registered at 0.22 (■), 0.99 (▲), 4.0 (▼) and 6.4 μs (♦) after laser pulse (λ_{ecc}= 355 nm). (b) Decay kinetic of **4**$^{+\bullet}$ recorded at 530 nm after the laser pulse (λ_{ecc}= 355 nm) in N_2-saturated MeCN; the full line represents the 1st order best fitting of the experimental data.

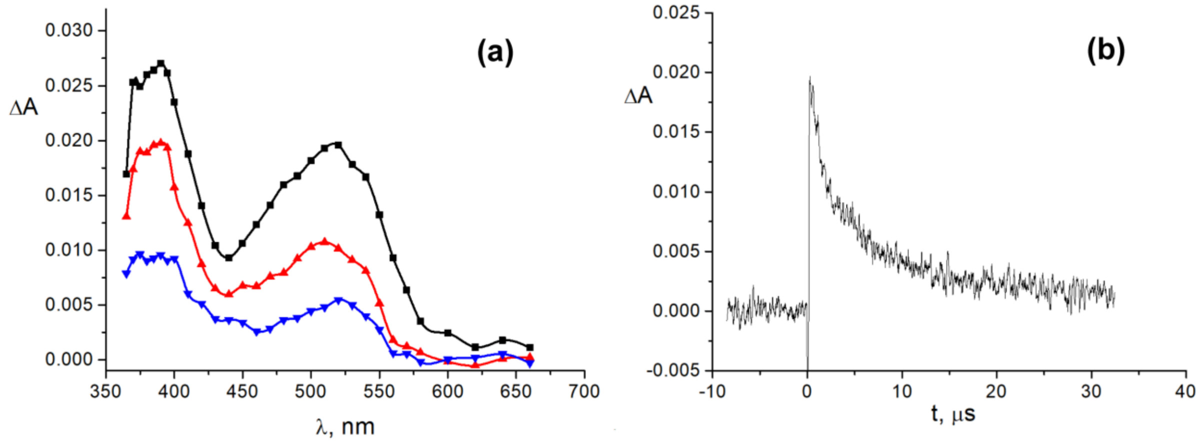

Figure S14. (a) Time resolved absorption spectra obtained in the photolysis of sulfoxide **5** (3.0×10^{-3} M) in the presence of 3-CN-NMQ$^+$ ClO_4^- (0.5×10^{-4} M) and toluene (1 M) in N_2-saturated MeCN at 22 °C registered at 0.30 (■), 2.4 (▲) and 7.6 μs (▼) after laser pulse (λ_{ecc}= 355 nm). (b) Decay kinetic of **4**$^{+\bullet}$ recorded at 520 nm after the laser pulse (λ_{ecc}= 355 nm) in N_2-saturated MeCN.

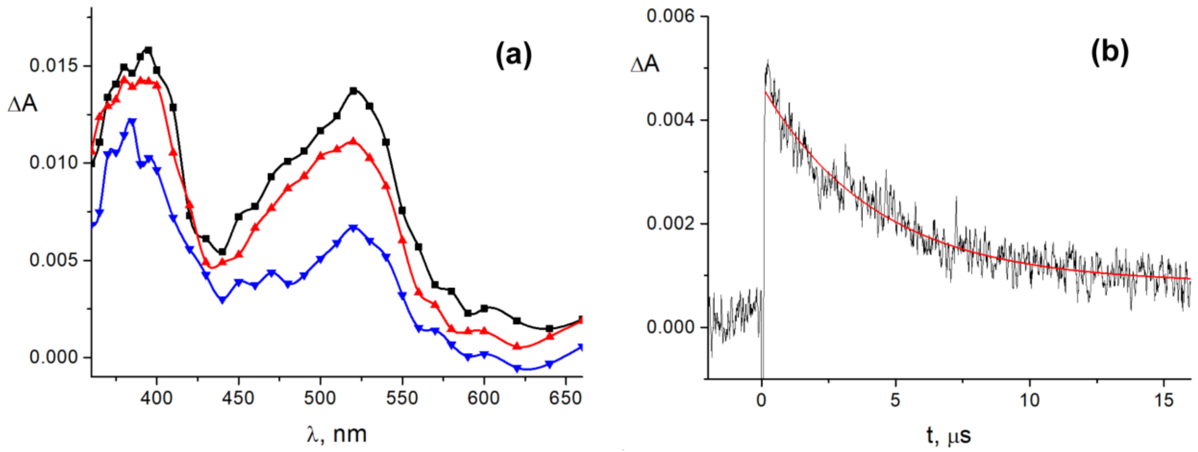

Figure S15. (a) Time resolved absorption spectra obtained in the photolysis of sulfoxide **6** (1.0×10^{-2} M) in the presence of 3-CN-NMQ$^+$ ClO_4^- (1.0×10^{-3} M) and toluene (1 M) in N_2-saturated MeCN at 22 °C registered at 0.13 (■), 0.88 (▲) and 6.4 μs (▼) after laser pulse (λ_{ecc}= 355 nm). (b) Decay kinetic of **6**$^{+\bullet}$ recorded at 530 nm after the laser pulse (λ_{ecc}= 355 nm) in N_2-saturated MeCN; the full line represents the 1st order best fitting of the experimental data.

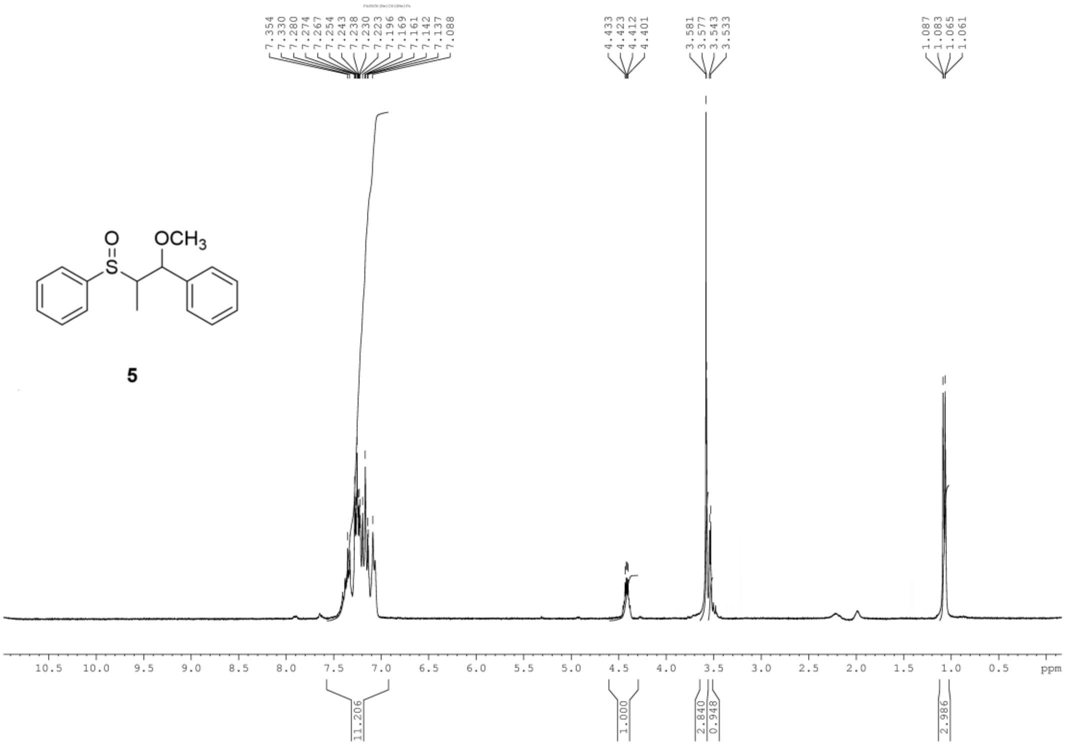

Figure S16. ^{1}H NMR (300 MHz) spectrum of **5** in $CDCl_3$.

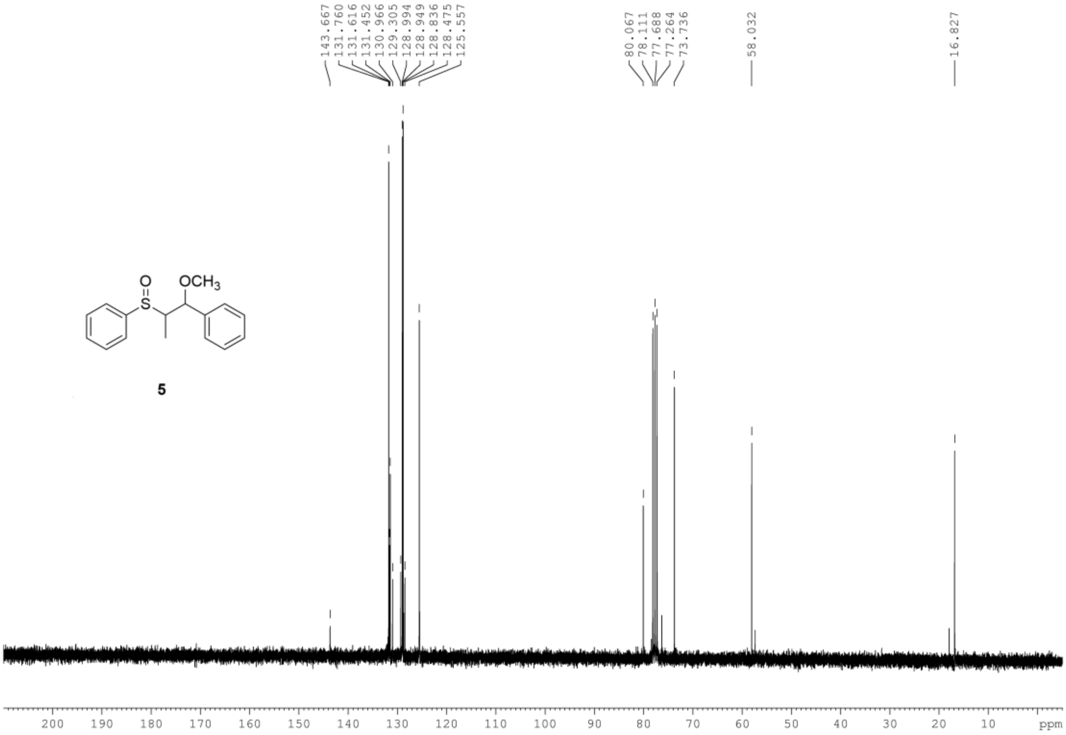

Figure S17. ^{13}C NMR (75 MHz) spectrum of **5** in $CDCl_3$.

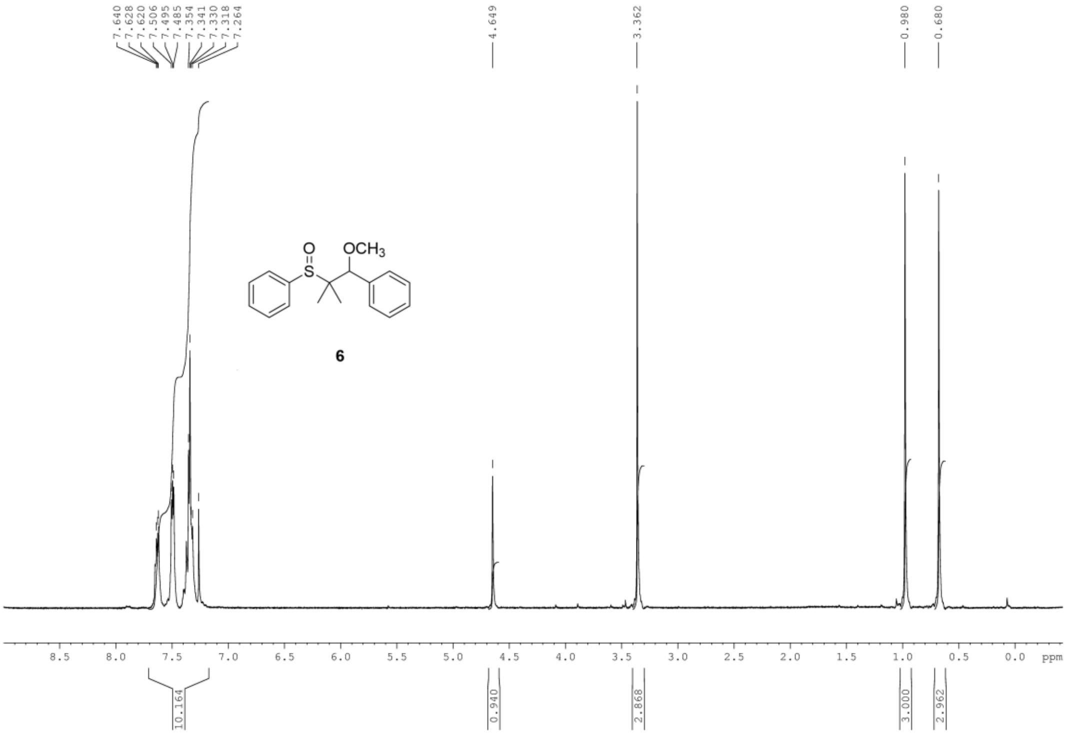

Figure S18. ^{1}H NMR (300 MHz) spectrum of **6** in $CDCl_3$.

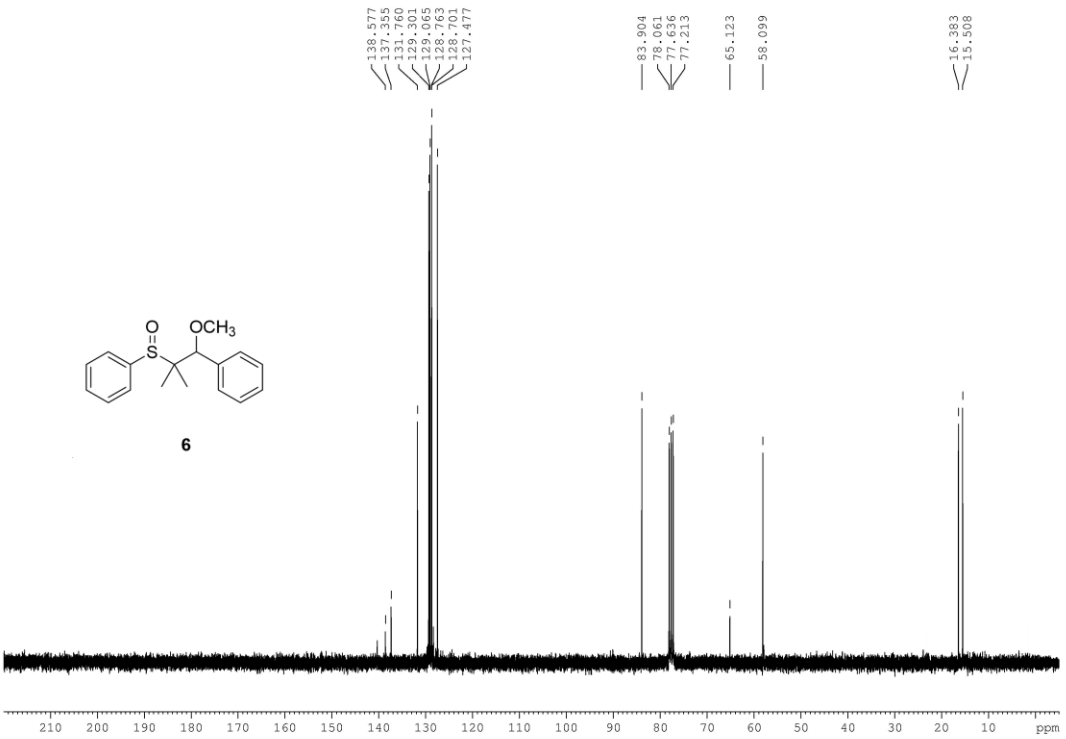

Figure S19. ^{13}C NMR (75 MHz) spectrum of **6** in $CDCl_3$.

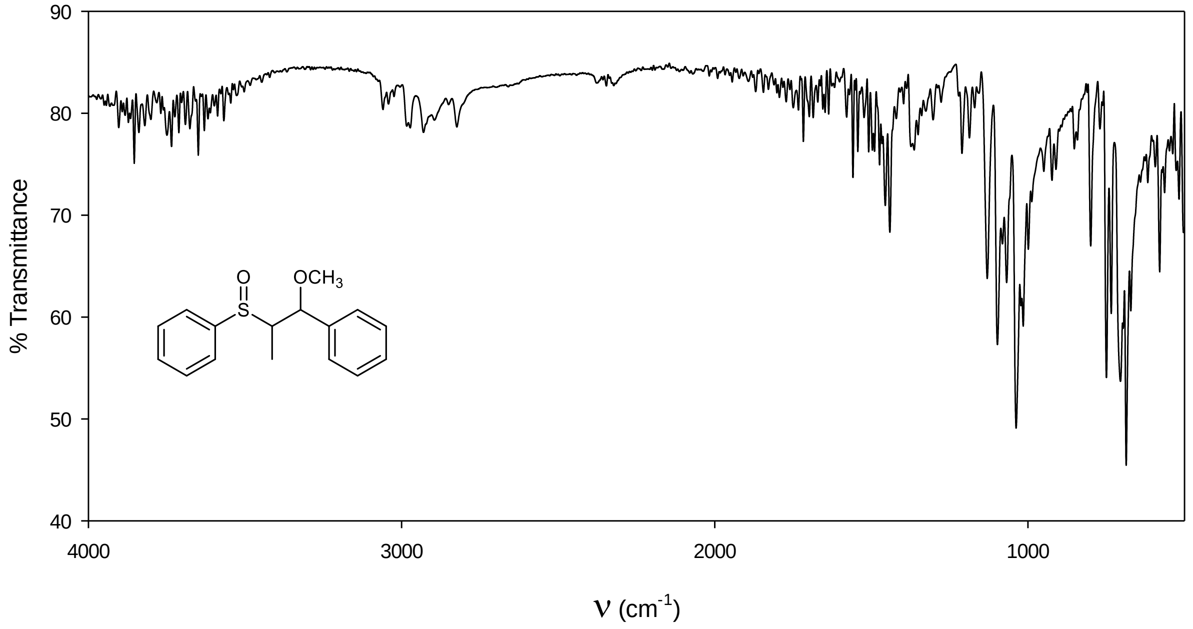

Figure S20. FT-IR spectrum of **5** (pure solid).

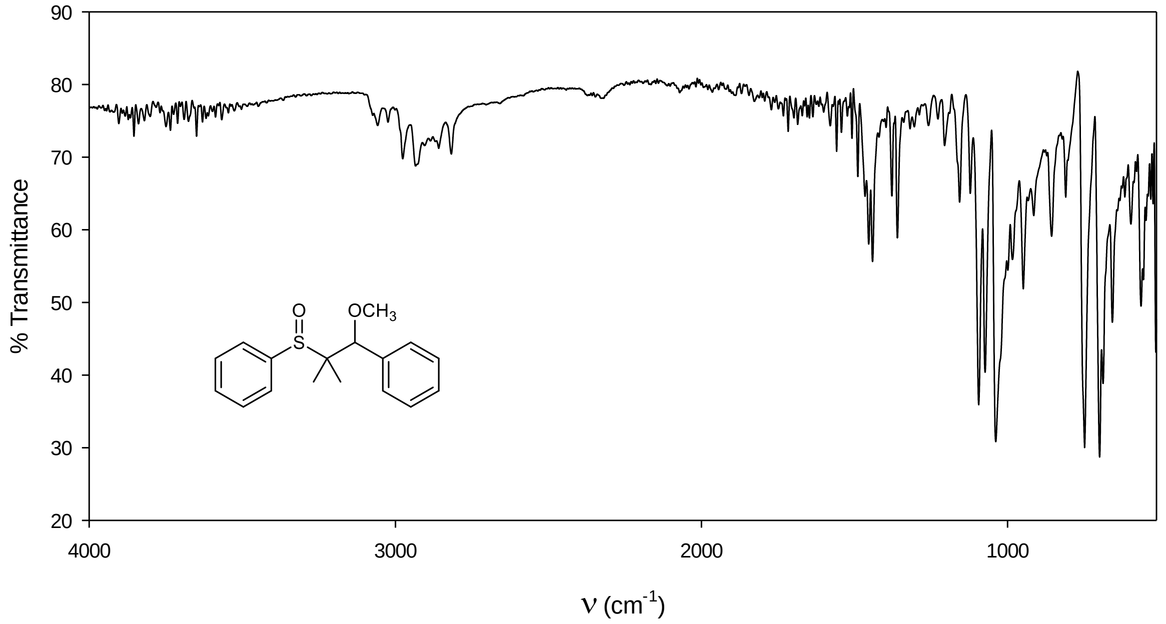

Figure S21. FT-IR spectrum of **6** (pure solid).
